# Enhanced mutualistic symbiosis between soil phages and bacteria with elevated chromium-induced environmental stress

**DOI:** 10.1186/s40168-021-01074-1

**Published:** 2021-06-28

**Authors:** Dan Huang, Pingfeng Yu, Mao Ye, Cory Schwarz, Xin Jiang, Pedro J. J. Alvarez

**Affiliations:** 1 Key Laboratory of Soil Environment and Pollution Remediation, Institute of Soil Science, Chinese Academy of Sciences, Nanjing, 210008 People’s Republic of China; 2 University of Chinese Academy of Sciences, Beijing, 100049 People’s Republic of China; 3 Department of Civil and Environmental Engineering, Rice University, Houston, 77005 USA

**Keywords:** Soil virome, Phage–bacterium interactions, Lysogenic conversion, Auxiliary metabolic genes, Chromium stress

## Abstract

**Background:**

Microbe–virus interactions have broad implications on the composition, function, and evolution of microbiomes. Elucidating the effects of environmental stresses on these interactions is critical to identify the ecological function of viral communities and understand microbiome environmental adaptation. Heavy metal-contaminated soils represent a relevant ecosystem to study the interplay between microbes, viruses, and environmental stressors.

**Results:**

Metagenomic analysis revealed that Cr pollution adversely altered the abundance, diversity, and composition of viral and bacterial communities. Host–phage linkage based on CRISPR indicated that, in soils with high Cr contamination, the abundance of phages associated with heavy metal-tolerant hosts increased, as did the relative abundance of phages with broad host ranges (identified as host–phage linkages across genera), which would facilitate transfection and broader distribution of heavy metal resistance genes in the bacterial community. Examining variations along the pollutant gradient, enhanced mutualistic phage–bacterium interactions were observed in the face of greater environmental stresses. Specifically, the fractions of lysogens in bacterial communities (identified by integrase genes within bacterial genomes and prophage induction assay by mitomycin-C) were positively correlated with Cr contamination levels. Furthermore, viral genomic analysis demonstrated that lysogenic phages under higher Cr-induced stresses carried more auxiliary metabolic genes regulating microbial heavy metal detoxification.

**Conclusion:**

With the intensification of Cr-induced environmental stresses, the composition, replication strategy, and ecological function of the phage community all evolve alongside the bacterial community to adapt to extreme habitats. These result in a transformation of the phage–bacterium interaction from parasitism to mutualism in extreme environments and underscore the influential role of phages in bacterial adaptation to pollution-related stress and in related biogeochemical processes.

**Video Abstract**

**Supplementary Information:**

The online version contains supplementary material available at 10.1186/s40168-021-01074-1.

## Background

Phages, bacterial viruses that constitute the most abundant and genetically most diverse biological entities in the biosphere, are critical components of microbial communities [1, 2]. The rapid development of viral metagenomics has advanced our understanding of phage abundance and composition in various ecosystems [3–7], allowing us to identify their functional importance more comprehensively. The sheer abundance and diversity of phages, together with their multifaceted impacts on microbial hosts, strongly indicate that phages can influence entire macroscopic ecosystems by affecting the native microbial communities [8–12]. However, previous virome studies mainly focused on phage species and genetic profiles from different ecosystems, while phage–bacterium interactions have been heretofore relatively understudied [5, 13]. To better understand the ecological and evolutionary roles of phages in ecosystems, it is important to elucidate phage–bacterium interactions and their response to environmental stresses.

Phages are obligate intracellular bacterial parasites who rely on the presence of appropriate hosts to persist and propagate [14, 15]. The classical view of phage–bacteria parasitism holds that phages propagate at the expense of their bacterial hosts and redirect host resources to produce virions [16]. Beside this parasitic pattern often observed under favorable conditions, mutualistic phage–bacteria interactions also occur under unfavorable circumstances (e.g., solar radiation or nutrient limitation) [17, 18]. Specifically, lysogenic phages can promote horizontal gene transfer by lysogenic conversion or transferring packaged bacterial genes to new hosts, which may expand the host metabolic profile and enhance microbial environmental adaptability [11, 19–22]. In return, the phages that are integrated into the host genomes in dormant forms (i.e., prophages) can take advantage of microbial environmental resistance to avoid direct exposure to harsh environments [23]. Typically, the extent of phage–bacteria mutualism is positively correlated with the abundance of lysogenic phages in a virome, lysogenized bacteria in a microbiome, and auxiliary metabolic genes (AMGs) carried by lysogenic phages [19].

Heavy metal contamination of soil poses a serious threat to public health and ecological security due to its widespread occurrence, persistence, and ecotoxicity [24, 25]. Heavy metal-contaminated soils generally exhibit a decreasing geochemical gradient from the contamination source [26, 27]. Such soils are recognized as ecosystems under complex stresses and are both relevant and convenient to study the interplay between phages, bacteria, and environmental stressors. However, the viromes in heavy metal-contaminated soils remain poorly characterized, and the response of phage-bacterium interactions to heavy metal-induced environmental stress is largely unknown. This critical knowledge gap should be addressed to inform microbiome adaptation to hostile environments and the role of phages in related biogeochemical processes. Advancing understanding of phage–bacterium interactions under different heavy metal stress may also help better assess the ecotoxicity of heavy metals and potential of microbiome engineering for contaminated soil remediation.

To this end, the objective of this study was to explore the variability of soil viromes and phage–bacterium interactions along gradients of environmental stress. Chromium (Cr)-contaminated sites were chosen to represent heavy metal soil contamination, due to the high frequency of occurrence and well-recognized ecotoxicity [28, 29]. Soil samples were taken along Cr gradients in one lightly polluted site (Luzhou, LZ for short) and one heavily polluted site (Zhangye, ZY for short). Metagenomic analyses for total environmental DNA and viral DNA were separately conducted to inform indigenous bacterial and viral profiles. Bacteria–phage linkages, commonality of lysogenicity within the virome, abundance of phage-carried AMGs and the lysogenized fraction of bacteria were investigated to demonstrate how variations in the virome and its functions were related to the indigenous bacteria under Cr-generated environmental stress. This metagenomic study reveals an enhanced mutualistic symbiosis between phages and bacteria with elevated Cr-induced environmental stress and highlights the roles of phages in mediating microbiome adaptation to hostile environments.

## Results

### Bacterial community profiles of Cr-contaminated sites

**Fig. 1:**
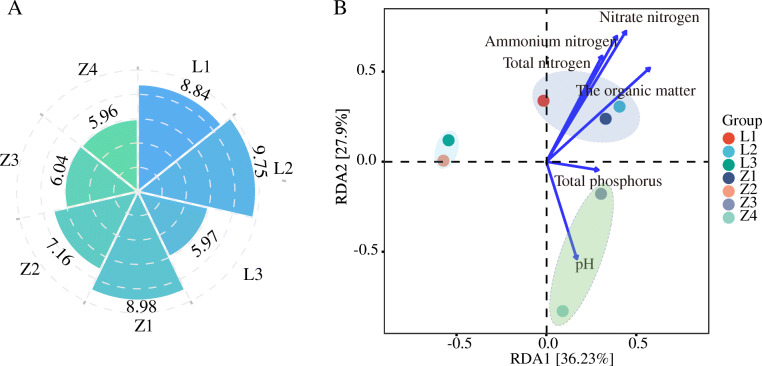
Profile of bacterial communities in Cr-contaminated soils. **A** The biomass of bacteria in Cr-contaminated soil. The petal lengths of the rose represent the value of log (copies/g soil) quantified by fluorogenic quantitative PCR reactions with 16S rRNA gene, which generally decreased with the increase of Cr contamination in both sites. **B** Redundant analysis (RDA) of bacteria in different soil samples. The results showed nitrate, ammonium, total nitrogen, organic matter and total phosphorus had the closest correlation with the composition of bacteria. The bacterial communities of each sample clustered most closely with samples under similar geochemical conditions. Specifically, L1, L2 and Z1 with low available Cr concentration were more similar, and L3 and Z2 with slightly higher pollution levels were clustered together. Z3 and Z4 were dramatically different from those in other sites

There was considerable variability in physicochemical and nutritional properties of the contaminated soil, including gradients in Cr contamination (Fig. S1 and Table S1). Based on the Cr pollution level and nutrient conditions, these seven samples can be roughly divided into three groups: a slightly contaminated group (L1, L2, and Z1 with available Cr concentrations of 0.11, 0.27, and 0.91 mg/kg, respectively), a moderately contaminated group (L3 and Z2 with Cr concentrations of 6.76 and 6.09 mg/kg, respectively) and a highly contaminated group (Z3 and Z4 with Cr concentrations of 413.84 and 465.42 mg/kg, respectively). Accordingly, there was a logical decrease in microbial biomass with the increase of Cr contamination and decrease in nutrient availability. The soil bacterial abundance, quantified by 16S rRNA abundance, fell dramatically by several orders of magnitude along the increasing Cr concentration gradient, from 9.75 to 5.97 log (copies)/gdw (per gram dry weight) in LZ, and from 8.89 to 5.96 log (copies)/gdw in ZY (Fig. 1A).

Metagenomic analysis of species richness, dominant bacterial species and beta diversity revealed a significant difference in the bacterial communities of soils with different levels of Cr pollution. Specifically, the Chao1 and ACE index showed that the increase in soil Cr concentration adversely affected the species richness of microflora (Fig. S2). Moreover, phylogenetic analysis and heatmap of the 565 dominant species (with a relative abundance of > 0.1%) in LZ and ZY sites showed that the bacterial species from the comparable contamination levels (slight, moderate, and severe) were more closely related, while those from different contamination levels differed observably (Fig. S3), although they were all mainly from *Proteobacteria* and *Actinobacteria*. Consistently, redundant analysis (RDA) of bacterial communities showed that bacterial profiles in L1, L2, and Z1 with low available Cr concentration were more similar, and those in L3 and Z2 with moderate pollution levels were clustered together. The bacterial communities in Z3 and Z4 were dramatically different from those in other sites, possibly due to the severe degree of pollution and associated poor nutritional status (Fig. 1B).

### Virome patterns along Cr contamination gradients

**Fig. 2:**
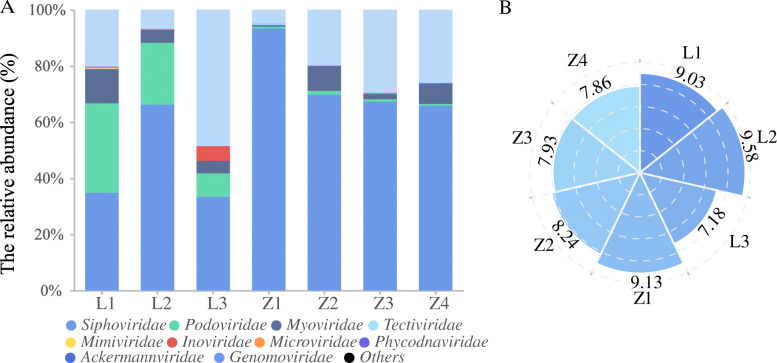
Profile of viral communities in Cr-contaminated soils. **A** The composition of the viral community at the family level from seven different locations at two Cr-contaminated sites shows that viral populations between these 7 different locations exhibited distinct profiles. **B** The abundance of viral particles in Cr-contaminated soil counted by fluorescence microscope. The petal lengths of the rose represent the abundance of viral particles per gram of soil (log (VLP/g soil)), which decreased with the increase of Cr contamination in both sites

In total, 5099 viral clusters (VCs) were identified from the 7257 viral contigs (> 5 kb) originating from site LZ and 3021 VCs were identified from the 4561 viral contigs (> 5 kb) originated from site ZY (Fig.S4A & B). As with most characterized viromes in various habitats [13, 30], the annotated phages in Cr-contaminated soil predominately belonged to *Siphoviridae*, *Podoviridae*, and *Myoviridae* families (Fig. 2A). The relative abundance of *Siphoviridae*, *Podoviridae*, and *Myoviridae* families among the seven different soil samples was 33.3–93.4%, 0.7–31.9%, and 0.5–12.2%, respectively (Fig. 2A). Similar to the trend of bacterial abundance, the abundance of viral-like particles (VLP, determined by fluorescence microscopy imaging) decreased from 9.58 to 7.18 log (VLP)/gdw in site LZ and from 9.13 to 7.86 log (VLP)/gdw in site ZY with the increase of Cr contamination (Fig. 2B).

Consistent with the trends observed in the bacterial communities, the phage populations among these seven different locations were significantly affected by soil Cr contamination and soil nutrient status. Cluster analysis of the top 20 viruses (with a relative abundance of > 1.0%) showed that viruses from samples with similar physical-chemical properties were more closely related: L1, L2 and Z1clustered together; Z1, Z2, and L3 clustered together; and Z3 was most closely clustered to Z4 (Fig. S5). RDA corroborated the viral composition patterns with virome of soil with the same level of Cr contamination clustered together (Fig. S6).

**Fig. 3:**
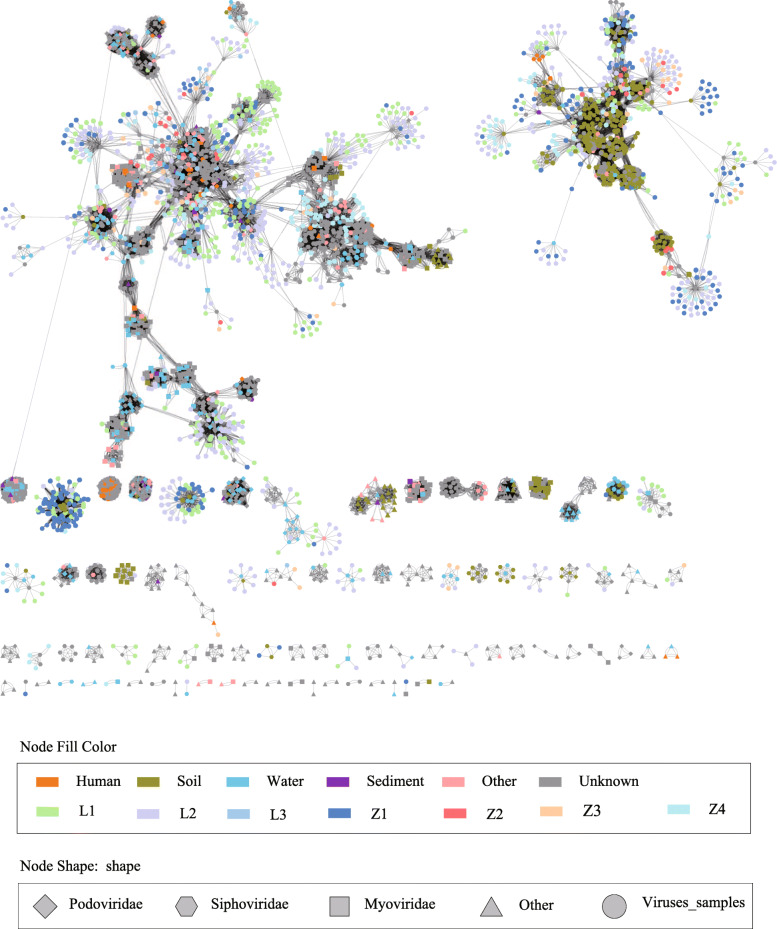
Relating viral contigs from Cr-contaminated soils to known viral sequence space. Viral contigs recovered from Cr-contaminated soils were clustered with all RefSeq (v 75) viral genomes or genome fragments with genetic connectivity to these data. Each node colors indicate sample and reference viral sequences’ habitat of origin. The edges (lines) between the nodes represent statistically weighted pairwise similarity scores of ≥ 1. The results show that viromes at different sites along a Cr-contaminated soil gradient have distinct specificity. And only a few of recovered viral contigs were associated with viruses recorded in NCBI databases (RefSeq, v 75) from soil, water, human, and sediment habitats

Compared with the prokaryotic viruses in the viral database (RefSeq, version 75) [31], the viromes of Cr-contaminated sites were highly novel and variant in terms of genetic profiles (Fig. 3). Specifically, approximately 92.3% of viral contig sequences are not related to RefSeq virus sequences. Only 7.7% of the viral contigs had high genetic similarity to viruses recorded in RefSeq (weighted pairwise similarity scores of ≥ 1). Whereas 24.6% of the matched viral contigs in the contaminated soil were linked with viruses found in soil, the values decreased from 38.0 to 20.9% with the increase of pollution concentration in each sample (Fig. S7). Consistent with the similarities in viral profiles, the soil viral contigs with similar levels of Cr contamination were assigned with relevant habitats (Fig. S7). Interestingly, 14.6% of the matched viral contigs were linked with aquatic viruses, and there were less than 10.0% viral contigs linked with viruses from sediments, the human body, and other habitats.

### Variation in host-specific features

**Fig. 4:**
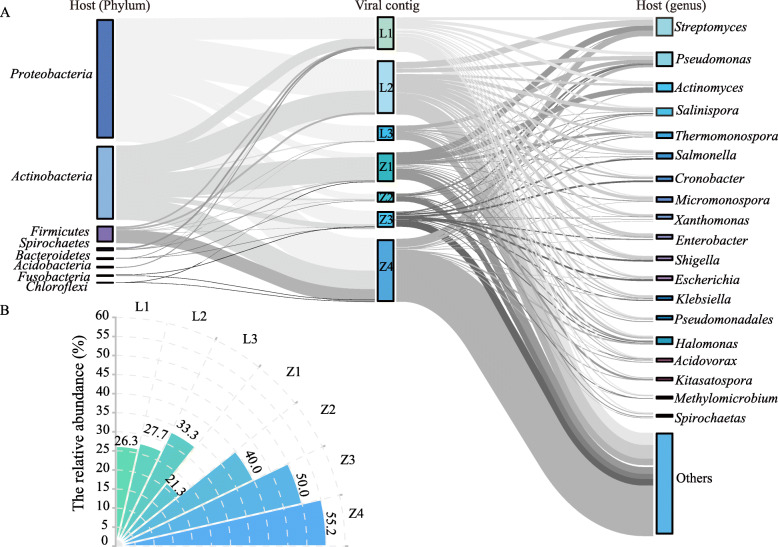
Predicted virus–host linkages in Cr-contaminated soils. **A** A total of 1044 hits were obtained between 646 phage contigs and 279 bacterial genera, and these host genera were mainly from 8 bacterial phyla. From left to right and the first columns show the distribution of bacterial hosts at the phylum level; the second column depicts viral contigs linking to hosts in the seven soil samples; the third column shows the distribution of the bacterial host assigned with CRISPR approaches at the genus level. The height of the column section represents the abundance of viruses and hosts. The results revealed that these viral host species predicted in the soil polluted by high Cr concentration mostly belong to the bacterial genera which have been reported to have strong adaptivity to better survive in heavy metal-polluted habitats. **B** The relative abundance of polyvalent phages within the viral communities of Cr-contaminated soils. Polyvalence was found to be consistently higher in soil samples with higher available Cr concentrations

The host spectra of viruses were estimated via matching CRISPR spacers in the IMG/VR database to viral contigs (Fig. 4). A total of 1044 hits were obtained between 646 phage contigs and 279 bacterial genera. Following the typical parasite–host pattern, the matched 279 host genera were mainly from eight bacterial phyla (Fig. 4A & Fig.S8A), which were comprised of the most abundant phyla in the bacterial community (Fig. S8B). For example, the phage contigs linking to *Proteobacteria* and *Actinobacteria*, the most abundant phyla (36.4% and 33.0%) in the bacterial community, accounted for 42.4% and 34.3% of the total phyla, respectively. Whereas most soil viruses (409 contigs) seemed to be specific with contigs linked to one specific bacterial genus, a considerable fraction of viruses (237 contigs) may have broad host ranges with phage contigs linked to more than two bacterial genera. The relative abundance of the possibly polyvalent phages (having links to two or more distinct genera), which had higher fitness under high environmental stresses and low bacterial abundance [32, 33], was higher in soils with higher Cr levels (Kruskal-Wallis (W-K) test, *p* < 0.05). Specifically, the fractions of polyvalent phages in the LZ site increased from 26.3% in L1 to 33.3% in L3, and their fractions in the ZY site increased from 21.3% in Z1 to 55.2% in Z4 (Fig. 4B). In Cr-contaminated soil, the top ten bacterial genera infected by polyvalent phages were *Pseudomonas*, *Salmonella*, *Cronobacter*, *Enterobacter*, *Escherichia*, *Klebsiella*, and *Shigella* from *Proteobacteria* phylum, and *Salinispora*, *Micromonospora*, and *Actinomyces* from *Actinobacteria* phylum.

In the low- and medium-contaminated sites, the relative abundance of *Pseudomonas*, *Cronobacter*, *Klebsiella*, and *Halomona* genera within the bacterial community increased with the available Cr concentrations in soil (Fig. S9). These four bacterial genera are characterized by their biofilm formation capabilities, low membrane permeability, and strong efflux pump systems conducive to bacterial survival under heavy metal stress [34, 35]. Accordingly, the relative abundance of phages linking to these four genera increased from 3.6–13.9% in the low contamination sites (L1, L2, and Z1) to 27.3–40.3% in the medium contamination sites (L3 and Z2) (Fig. 4 & Fig.S9). However, the dominant bacterial genera linked by phage in Z3 and Z4 with high Cr levels (413.8 and 465.4 mg/kg) were significantly different from those in the previous slightly and moderately contaminated sites. The dominant genera of Z3 included *Micropruina*, *Brevibacterium*, *Acidithiobacillus*, and *Zoogloea*, and that of Z4 included *Bacillus*, *Stenotrophomonas*, *Comamonas*, and *Lysinibacillus*. These bacteria are often detected in heavy metal-contaminated environments because of their strong resilience and adaptivity to high levels of salts and heavy metals [36–38]. Overall, the host–phage linkage corroborated phages as bacterial parasites depending heavily on the appropriate host flora to persist in Cr-contaminated soils.

### Variation in the relative abundance of lysogenic phage indicators (LPIs)

**Fig. 5:**
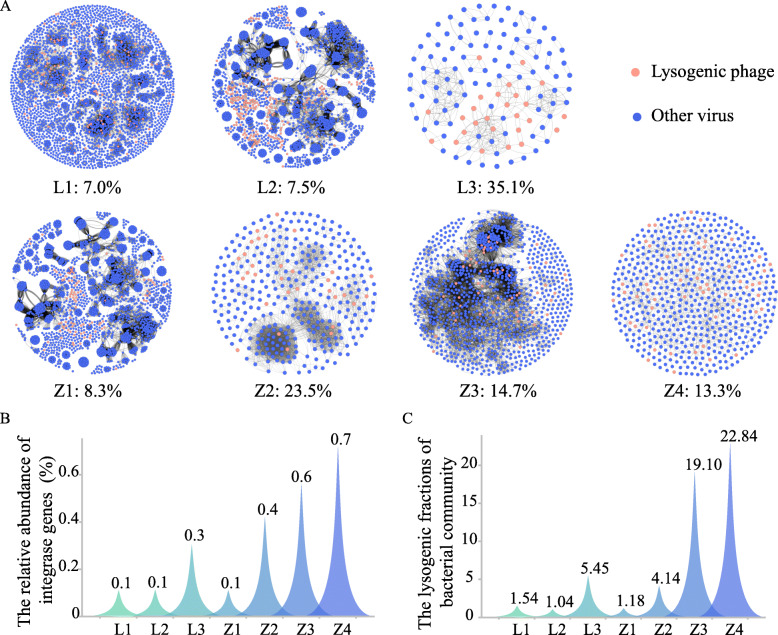
Lysogen occurrence within the soil virome along Cr concentration gradients. **A** Co-occurrence networks of viral contigs in seven soil samples. Nodes (circles) represent viral contigs. Orange represent the contigs containing lysogenic phage indicators; dark blue nodes are all other viral contigs. Shared edges (lines) between nodes indicate a correlation, and the more edges in a node indicate a closer genetic relationship between contigs. The values to the right of each sample name are the relative abundances of lysogenic phages within the corresponding sample. As the distance between sampling location and source of contamination grew, the relative abundance of lysogenic phages decreased in the lightly polluted LZ site. In the heavily polluted ZY site, an increasing trend from sample Z1 to Z2 was observed, which then showed a decreasing trend between Z2 and Z4. The relative abundance of phage integrase genes found in bacteria (**B**) and inducible lysogenic fractions (**C**) at the sampling locations consistently increased in tandem with Cr concentrations

The annotation of viral genomes was performed by comparing the predicted proteins against the PFAM database (33.1) to explore the lysogenicity of virome. Viral contigs with integrase genes (Table S2) were counted as lysogenic phages [39, 40]. The relative abundance of lysogenic phages increased with Cr levels in the LZ site: L1 (7.0%) < L2 (7.5%) < L3 (35.1 %) (Fig. 5A). For ZY, the relative abundance of the lysogenic phage formed a bell-shaped curve as the Cr level increased. The relative abundance of lysogenic phages increased from Z1 (8.3%) to Z2 (23.15%), while decreased in Z3 (14.7%) and Z4 (13.3%) (Fig. 5A). There was a significant increase in the relative abundance of viral integrase genes in bacterial genomes in response to the increased available Cr levels (W-K test, *p* < 0.01): L1 (0.1%) ≈ L2 (0.1%) < L3 (0.3%) in the LZ site, and Z1 (0.1%) < Z2 (0.4%) < Z3 (0.6%) < Z4 (0.7%) in the ZY site (Fig. 5B). Furthermore, more phages could be chemically induced from bacteria in more contaminated soil, which corroborated the host genome that contained more prophage as Cr levels increased (W-K test, *p* < 0.05). In L1, L2, and Z1, the lysogenic fractions were all lower than 2 VLP/cell, while the lysogenic fractions increased to 5.45 and 4.14 VLP/cell in L3 and Z2, respectively. The corresponding fractions further increased to 19.140 and 22.84 VLP/cell in Z3 and Z4, respectively (Fig. 5C).

### Community-wide comparative genetic profiles of viromes

The annotation of viral genomes was performed by comparing the predicted proteins against the Non-supervised Orthologous Groups (eggNOG) and Kyoto Encyclopedia of Genes and Genomes (KEGG) databases. Nonmetric multidimensional scaling (NMDS) analysis of viral genomes revealed that the viral contigs from different locations were more similar in functional composition when the spatially distinct locations had similar Cr levels (Fig. S10). Specifically, the low Cr level samples (i.e., L1, L2, and Z1) clustered together, medium Cr level samples (L3 and Z2) clustered together, while high Cr level samples (i.e., Z3 and Z4) but distinct from the other samples.

Moreover, viruses carried multiple AMGs related to host metabolism, and their relative abundance increased significantly with available Cr concentrations in slightly and moderately contaminated sites (Fig. S11). For example, the relative abundance of genes related to energy production and conversion (Group C) and lipid transport and metabolism (Group I) increased within the virome with available Cr concentrations increased from 0.11 to 6.97 mg/kg. However, at severe pollution locations (Z3 and Z4), the relative abundance of these genes in virome decreased significantly compared with Z2. Similar trends were observed for genes related to inorganic ion transport and metabolism (Group P); secondary metabolite biosynthesis, transport, and catabolism (Group Q); intracellular trafficking, secretion, and vesicular transport (Group U); and defense mechanisms (Group V). These genes are well recognized for mediating host transport and ion secretion. Upregulation of these genes is generally beneficial to microbial energy conversion under environmental stress, and to enhance detoxification processes (e.g., efflux and chromate reduction) [41, 42].

**Fig. 6:**
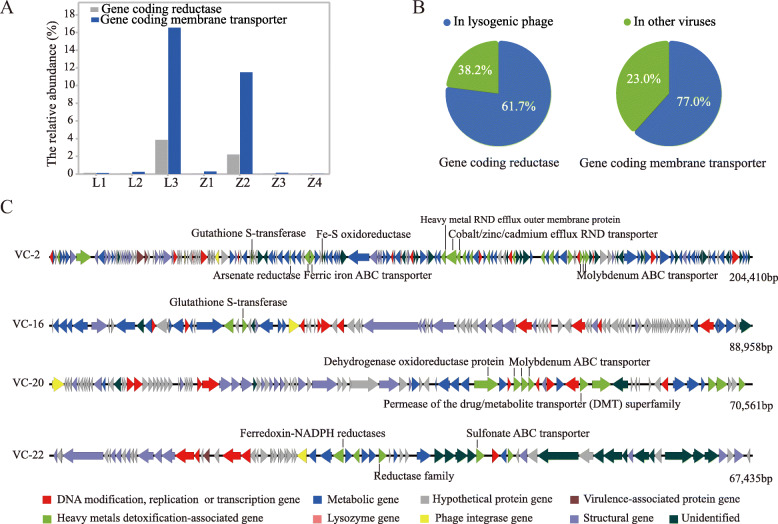
Genomic analysis of viral genomes in Cr-contaminated soils. **A** The relative abundance of viral genes coding membrane transporter (dark blue) and reductase (gray) were annotated by KEGG datasets (Table S3 and Table S4). There was a significant increase in the relative abundance of these genes associated with reduction and transport of heavy metals in L3 and Z2. **B** The distribution ratio of genes coding reductase (left) and membrane transporter (right) on lysogenic phages (blue) and other viruses (green), which were are calculated by determining whether these genes are carried by viral contigs with the integrase genes. It shows that over 60% of them were on lysogenic phages, so lysogenic phages are an important reservoir for MRGs. **C** Genome map of four viral contigs containing both the phage integrase gene and the metal detoxification gene. Key of gene families and their colors are found in the figure, and arrow length within the gene maps correspond to the size of the open reading frame (ORF). Detailed function descriptions of the seven viral contigs are listed in Table S5

Bacterial Cr detoxification mainly involves efflux of intracellular Cr (VI) ions through membrane transporters and enzymatic reduction of Cr (VI) into less toxic Cr (III) [43, 44]. KEGG databases were used to screen functional genes corresponding to microbial heavy metal detoxification (i.e., membrane transporter gene (Table S3) and reductase gene (Table S4) in viromes. As shown in Fig. 6A, the relative abundance of reductase genes in viromes increased from 0.03 to 3.8%, and membrane transporter increased from 0.1% to 16.5% as the concentrations of available Cr increased from 0.11 to 6.76 mg/kg, which demonstrated that phages can be important reservoirs of bacterial heavy metal resistance genes (MRGs) in contaminated sites. However, the relative abundance of bacterial MRGs in Z3 and Z4 decreased to 0.2% and < 0.1%, respectively. Notably, over 77.0% of the genes encoding membrane transporters and 61.7% of reductases were located on lysogenic phages (Fig. 6B), by investigating whether these genes are carried by viral contigs with the integrase genes. This suggests that lysogenic phages may serve as carriers of MRGs in highly contaminated sites.

### Case study of lysogenic phages with MRGs

To corroborate that putative lysogenic phage-encoded AMGs (especially MRGs), four viral contigs (VC-2, VC-16, VC-20, VC-22) with integrase gene were randomly selected from the viromes as representatives and their genomic profiles were systematically analyzed. All VCs carried prokaryotic genes associated with heavy metal detoxification (Fig. 6C). Notably, viral contig VC-2 encoded genes including Arsenate reductase, Glutathione S-transferase, Ferric iron ABC transporter, Fe-S oxidoreductase, Heavy metal RND efflux outer membrane protein, Cobalt/zinc/cadmium efflux RND transporter, and Molybdenum ABC transporter. Viral contig VC-20 encoded Dehydrogenase oxidoreductase protein and Permease of the drug/metabolite transporter (DMT) superfamily. For VC-22, there were genes encoding Ferredoxin-NADPH reductases, Sulfonate ABC transporter, and Reductase.

## Discussion

Phages, being the most common biological entity on earth, are a great untapped resource with possible applications in medical, industrial, and agricultural fields [45]. To identify the ecological and evolutionary role of phages in the biosphere and reveal the tactics of microbial adaptivity to hostile environments [46, 47], we systematically investigated variations of the viral community profile and phage–bacterium interactions along horizontal gradients of Cr-induced environmental stresses at two contaminated sites. The abundance and composition of viral communities varied greatly alongside their host communities across contamination and geochemical gradients. As Cr-induced environmental stresses increased, phage communities exhibited an enhanced beneficial relationship with the bacterial communities that is reflected by elevated relative abundance of lysogenic phages in the viromes and higher lysogenic fractions of bacteria. Furthermore, viral genomic analysis showed that phages under higher Cr-induced stresses carried more AMGs that can contribute to microbial heavy metal detoxification and survival in hostile environments. These findings inform variations in interactions between phages and microbes under adverse conditions and advance our understanding of the contribution of phages in biogeochemical cycles.

As is common for heavy metal contamination sites, increased levels of Cr were associated with decreased nutrient availability at both sites LZ and ZY (Table S1). Accordingly, the toxicity of Cr and the accompanying change in trophic conditions significantly altered both soil microbial and viral communities (Figs. 1 & 2). Notably, viral and bacterial communities from soil samples with similar Cr contamination levels rather than spatial proximity had more similarities in composition and abundance of functional genes (Fig. 1B, Fig. S6, & Fig. S10). Interestingly, the viromes in Cr-contaminated soils were mostly not linked to known viruses recorded in the RefSeq database (Fig. 3), possibly due to the unique physical and chemical properties of Cr-contaminated sites compared with other characterized habitats (uncontaminated soil, surface water, human gut, and sediment) [48]. This suggests that viromes of metal-contaminated soils (and possibly other extreme environments) might represent an untapped gene reservoir of biological agents with unknown potential for medical and environmental applications.

Phages as obligate intracellular parasites rely on the presence of appropriate hosts to persist and propagate [14, 15], which is corroborated by a high degree of consistency between the predicted phage host and the dominant bacteria in soils (Fig. 4A, Fig.S8 & Fig.S9). Relative abundance of bacterial genera (e.g., *Micropruina*, *Brevibacterium*, and *Bacillus*) with strong heavy metal resistance and survival capability under adverse conditions [49] increased in heavily Cr-polluted environments; CRISPR-based host linkage data revealed a tandem increase in the relative abundance of phages dependent on these resistant hosts. From an evolutionary perspective, bacteria with stronger resistance will have higher biomass and more stable intracellular environments under adverse conditions, providing better survival and reproduction conditions for their corresponding phages [50]. Furthermore, broad host range phages which can use multiple hosts to propagate are enriched in nutrient-poor environments or in conditions of low host density [33]; this research corroborates this trend for the first time within a Cr-contaminated area showing that the polyvalent strategy is viable to enhance phage fitness under severe environmental stress (Fig. 4B).

Furthermore, the capacity and tendency of phages towards a lysogenic lifestyle were generally enriched when faced with elevated levels of heavy metal pollution (Fig. 5). This corroborated the theory that lysogeny is a highly refined relationship whose development may be closely related to growth in unfavorable or even hostile conditions [19, 30, 51]. Both computational and experimental analyses demonstrated that the closer to the source of contamination, the higher abundance of prophages within the genomes of bacterial members of the community (Fig. 5B & C). When faced with hostile environments, phages capable of entering lysogeny have shown a higher inclination to integrate into their host genome as a prophage, establishing a more mutualistic relationship [16].

The relative abundance of lysogenic phages within the free virome in highly contaminated samples (Z2, Z3, Z4, and L3) increased significantly above that of low contamination samples (Z1, L1, and L2) (Fig. 5A). Interestingly, when examining the ratio of lysogenic to lytic phages in the free virome, the extremely contaminated samples (Z3 and Z4), had lower relative abundances of lysogenic phages, decreasing to nearly one half of that for moderately contaminated Z2 at the same sampling site. To explain this drop, we posit that with further deterioration of the surrounding environment, lysogenic phages may transform into prophages and lurk in the host body [52], which was corroborated by the increase of prophage in bacterial genome (Fig. 5B & C). Therefore, the number of free but lysogenic capable phages in the ultra-harsh environment would decrease. These findings inform the potential preference of lysogenic viruses in more extreme environments, suggesting that the lysogeny should be favorable for phages under heavy metal pollution stress. However, it should be noted that the host spectra estimated via the CRISPR-based approach may need further verification by other host-prediction models [53] or combined experimental and computational approaches [54].

Previous studies reported that phage-carried AMGs can contribute to the genetic and phenotypic evolution of their hosts and ultimately provide the hosts with diversified competitiveness and environmental adaptability [16, 52]. In our study, many MRGs encoding machinery for the detoxification of heavy metals from bacteria were detected in the virome (Fig. 6A & C). Our analysis also highlights a consistent trend between the relative abundance of MRGs and lysogenic phages within the virome (Figs. 5A & 6A). That is mainly because most MRGs are distributed on lysogenic phages (Fig. 6B). Therefore, when lysogenic phages integrate into host genomes in prophage form, the relative abundance of MRGs in the free virus particles also decreases. Lysogenic phages carrying AMGs (e.g., MRGs) suggest the potential of lysogenic phages to transfer beneficial genes in the microbial community and thus improve the survival ability of hosts in extreme environments [55]. Therefore, there is strong tendency toward mutualism relationship between phage and their hosts based on lysogenic integration with the further aggravation of Cr pollution in soil. In this relationship, lysogenized bacteria provide a more stable environment for phages [20], in return for which they may benefit from the expression of phage-carried AMGs [56–58]. Furthermore, the discovery of MRGs in the virome highlights the influence of viral communities on biogeochemical process in contaminated sites, which may inspire phage-based strategies (e.g., virome transplant) for microbiome engineering [60]. Given that phages are recognized as significant vectors of antibiotic resistance genes and pathogenic genes in various environments [11], the importance of bacteria–phage interactions in mediating bacterial resistance and possible pathogenesis may be underestimated, and their influence on public health warrants further investigation.

## Conclusion

This study of virome and metagenome interactions in soils subjected to various levels of stress associated with Cr contamination revealed that phage–bacterium interactions change from antagonistic (parasitic) at low stress towards a more mutualistic relationship with increasing Cr concentrations. This mutualistic symbiosis is mainly based on the lysogenic integration of lysogenic phages. That is, the lysogenic phages carrying MGRs can improve their host’s ability to resist toxicity of heavy metals by integrating themselves into the host genome, which in turn facilitates expression of resistance genes and reproduction of related phages. This demonstrates that viral populations and their functions have a profound influence on the adaptation of prokaryotic communities and their ability to withstand adverse geochemical conditions. In addition, efforts to remediate and restore heavy metal-contaminated sites could benefit from advanced understanding of interactions between bacterial and phages and their manipulations to enhance redox processes that reduce metal toxicity and mobility.

## Methods

### Site description and sampling

Seven soil samples were collected from two Cr residue-contaminated sites, located in Zhangye City (ZY, N 38° 25′ 49″ E 100° 48′ 41″) with relatively high chromium pollution and Luzhou City (LZ, N 28° 54′ 24″, E 105° 31′ 26″) with light chromium pollution, in September 2019. Soil samples are collected at intervals of 50 m, from the Cr-slag dump center towards the edge of the site. According to the site area, 3 soil samples (L1, L2, and L3) were collected in LZ, and 4 soil samples (Z1, Z2, Z3, and Z4) were collected in ZY. Each soil sample was a manually homogenized composite of five subsamples collected at the surface layer (5–10 cm) and sieved through a size 20 mesh. The details of sampling locations are shown in Fig.S1. The physical and chemical properties (i.e., pH, organic matter, total phosphorus, total sulfur, total potassium, available phosphorus, nitrogen, nitrate nitrogen) of soil samples were determined by standard methods [61]. The total Cr, available Cr (i.e., the total amount of water-soluble and exchangeable Cr), and hexavalent Cr concentrations were measured by ICP-MS (Agilent 7700x) according to the USEPA Method 7196A [62]. The concentrations of available Cr in the seven samples varied from 0.11 to 465 mg/kg (Table S1).

### Quantification of bacteria and virus in soil

Fluorogenic quantitative PCR reactions was conducted with 16S rRNA gene V4–V5 primer pairs to determine the biomass of bacteria in soil samples from ZY and LZ (Primer sequences (5’-3’) were 16S-515F: “GTGCCAGCMGCCGCGG” and 16S-907R: “CCGTCAATTCMTTTRAGTTT”). To determine the biomass of viruses in soil samples from ZY and LZ, the inverted fluorescence microscope (Nikon, eclipse Ti-S) was used to observe soil virus particles stained with SYBR Gold fluorescent dyes as previously described [63], and Fig. S12 is a typical image, in which viruses are the most numerous tiny dots, while bacteria are larger with definite shapes and edges.

### Total DNA extraction, metagenomic sequencing, and analysis

FastDNA Spin Kit for Soil (MP Bio, Irvine, CA) was used to extract total DNA from the soil samples according to the manufacturer’s instructions. DNA quantification was performed with a Qubit 3.0 fluorometer using the dsDNA system (Invitrogen, Waltham, MA). According to the Illumina TruSeq DNA Sample Preparation Guide, by using NEBNext Ultra II DNA Prep Kit (New England Biolabs, Ipswich, MA) and NovaSeq 6000 S4 Reagent Kit (300 cycles) (Illumina, San Diego, CA), genomic libraries were constructed and sequenced on Illumina Hiseq 4000 platform (2 × 150 bp, paired-end reads) at Shanghai Personal Biotechnology Co., Ltd. (Shanghai, China). Finally, 16-GB sequence data was obtained for each of the samples. The quality control of raw reads was conducted by Cutadapt (v1.17) and FASTP (0.20.0) [64], and high-quality reads were used to assemble sequences with MEGAHIT v1.0. Minimap2 [65], Samtools [66], and blast2lca [67] were used to complete the construction of non-redundant sequence sets. After predicting the open reading frame (ORF) in sequences using MetaGeneAnnotator [68], redundant sequences were removed based on 90% sequence similarity by using CD-HIT [69]. Soap.coverage (http://soap.genomics.org.cn/) was used to calculate contig abundance and gene TPM (transcripts per million) value using a script (https://github.com/EnvGen/metagenomics-workshop/blob/master/in-house/tpm_table.py). The functional annotation of predicted ORFs was performed through assigning predicted ORF sequences to the PFAM 33.1 [70], KEGG database [71], and eggNOG v5.0.0 with a cutoff of E value < 0.001 [72]. Taxonomic classification at the species level was annotated via the NCBI-NT database (v2016-6-19) using Kaiju with the default run mode Maximum Exact Matches [73].

### Virus particle elution and verification

To separate viral nucleic acid from bacterial nucleic acid, several measures were taken to extract and enrich particles from soil following previous protocols (Fig. S13) [74]. Briefly, fresh soil sample was suspended in 4 °C potassium citrate buffer (10 g/L C_6_H_5_K_3_O_7_, 1.92 g/L Na_2_HPO_4_·12H_2_O, and 0.24 g/L KH_2_PO_4_, pH = 7). After bath sonication in an ice–water mixture for 3 min at 47 kHz, the suspension was centrifuged (7000 rpm, 4 °C, 10 min) to obtain the virus-containing supernatant. This was subsequently followed by centrifugation and filtration (0.2 μm) to remove impurities larger than 0.2 μm [73]. It should be noted that this filtration process may exclude viruses larger than 0.2 μm, leading them not to be included in further analysis of the viral population. The virus particles in the filtrate were initially concentrated by tangential flow filter cartridge (TFF, Sartorius Vivaflow 50 30000MWCO PES) followed by precipitation by polyethylene glycol 8000. The obtained virus pellets were treated with DNase and RNase to remove free DNA and RNA before viral DNA extraction [75]. In addition, virus particles were verified by transmission electron microscope (Zeiss LSM710) with the phosphotungstic acid counterstaining method (Fig. S14) [76]. It should be mentioned that the enrichment method of virome in this study is not perfect in the acquisition of giant phages.

### Viral DNA extraction and metagenomic sequencing

Viral DNA was extracted using the AllPrep PowerViral DNA/RNA Kit (MP Bio) following the manufacturer’s instructions. The extracted DNA was subjected to whole genome amplification (KAPA HiFi HotStart ReadyMix) to meet the metagenomic sequencing requirements. Libraries were constructed before sequencing on Illumina Hiseq4000 platform as described in *“Total DNA extraction, metagenomic sequencing and analysis”*. The protocol of quality control and assembly of virome is also the same as above. It should be mentioned that the enrichment method of viral nucleic acid in this study is not perfect in the acquisition of RNA viral information.

### Viral contig identification, taxonomic classification, and functional gene annotation

To eliminate potential bacterial contamination, “Virsorter + vHMM” method was used for the identification and annotation of viral contig > 5 kb in length [14, 77]. According to previous reports, VirSorter can provide near-perfect identification (> 95% recall and 100% precision) [78], and vHMM demonstrates a specificity of 99.6% for viral detection with a 37.5% recall rate [14]. Specifics are as follows: firstly, Virsorter was used with default parameter values, and the categories 1, 2, 4, and 5 of Virsorter predictions were considered as viral contigs. Secondly, the protein sequences of the remaining contigs were scanned with vHMM pipeline under 3 criteria (refer to http://portal.nersc.gov/dna/microbial/prokpubs/EarthVirome_DP/): (a) the contigs had at least 5 hits to viral protein families, and the total number of genes covered with KO terms on the contig was < 20%; the total number of genes covered with Pfams ≤ 40%; and the number of genes covered with viral protein families > 10%; (b) the contigs were selected as viral contigs when the number of viral protein families on the contig were equal or higher than the number of Pfams (bacterial genes); (c) the contigs for which the number of viral protein families was equal or higher than 60% of the total genes were classified as viral contigs. Viral contigs must satisfy at least one of the above three criteria. Then, vcontact2 was used to annotate the taxa of these viral contigs [19]. The functional annotation of non-redundant proteins of viral contigs was performed through assignment of predicted proteins to the PFAM, KEGG, and EggNOG databases with a cutoff of E value < 0.001 [72].

### CRISPR-based phage–bacteria linkage

Clustered Regularly Interspaced Short Palindromic Repeats (CRISPR) were used to predict phage–bacteria linkage based on the sequence similarity between the spacers in microbial CRISPR regions and the protospacers in viral genomes [19]. The sequences of all viral contigs were aligned to the spacer sequences deposited in the IMG/VR database (https://img.jgi.doe.gov/vr/). Specifically, all viral contig and spacer sequences from the shared genomic regions were queried by BLASTn (E value 10^−10^ and 100% nucleotide identity) to obtain the information about the potential host of the virus and finally link the virus to its bacterial host.

### Prophage induction assay by mitomycin-C

Induction of prophages was performed as previously described [51]. Briefly, 1 g of each of the original soil samples were suspended in 9 ml sterilized deionized water, and shaken for 20 min at the maximum speed, 20,000 rpm. Then, these resuspensions were inoculated into sterile water-soluble organic carbon (WSOC) solution both with and without mitomycin-C (final concentration of 1 μg/ml). These cultures were incubated at room temperature, shaking at 200 rpm for 24 h under in the dark. To harvest the induced and free phages, the cultures were passed through a 0.2-μm sterile membrane. The filtrates were then treated with glutaraldehyde (final concentration was 2.5%) as a fixative and stored at 4 °C for 24 h prior to staining.

Staining and fluorescence counting were performed as previously described by Anand, et al. [63]. After counting, the number of phages induced from a single bacterium was calculated using the following formula:

$$ \mathrm{X}=\left({\mathrm{V}}_{\mathrm{i}}-{\mathrm{V}}_{\mathrm{ck}}\right)/B $$

Here, V_i_ is the abundance of viral-like particles in the treatment with mitomycin-C, V_c_ is the abundance of viral-like particles in control treatments, and B is the bacterial abundance in the corresponding soil sample (by fluorogenic quantitative PCR reactions).

### Data analysis

The data analysis and visualization process involved in this study were conducted by Excel 2013 and R ×64 3.6.2. In order to clarify the correlation of viral contigs recovered in Cr-contaminated soil and other known viruses, the gene similarity score between the acquired virus contigs and known bacterial and archaeal viruses (NCBI RefSeq, version 75) was calculated according to Trubl et al. [31] (Genome pairs with a similarity score of 1 were considered remarkably similar). Then Cytoscape3.7.1 (http://cytoscape.org) was used to visualize the co-occurrence network, using an edge-weighted spring-embedded model. The Maximum-likelihood phylogenetic tree and heat maps of dominant bacteria (relative abundance greater than 0.1%) were produced using MEGA7 with the JTT model and iTOL (https://itol.embl.de/). The RDAs of viruses and bacteria were performed using R packages ggbiplot to analyze the viral and bacterial composition and diversity of different samples. In addition, functional genes annotated in KEGG databases corresponding to microbial heavy metal detoxification (i.e., membrane transporter gene (Table S3) and reductase gene (Table S4)) in the metagenome and virome were isolated to determine the detoxification potential of the microbial community. The differences between genetic profiles of viral communities were assessed by NMDS analysis utilizing R package vegan [79]. Based on PFAM databases, phage integrase family genes (as listed in Table S2) in the metagenome and virome were screened out to investigate changes in the relative abundance of lysogenic phages [29]. The statistical significance of differences at different Cr-contamination levels (i.e., the lysogenicity fraction of bacterial community, the relative abundance of MRGs, lysogenic phages, and polyvalent phages among soils with different pollution levels) were determined at the 95% confidence level (*p* < 0.05) using the non-parametric Kruskal-Wallis test (K-W test) [30].

## Supplementary Information

**Additional file 1: Table S1.** Physical chemical parameters of the soil samples. **Table S2.** Integrase family genes (annotated using PFAM). **Table S3.** Membrane transporter genes (annotated using KEGG). **Table S4.** Reductase genes (annotated using KEGG). **Table S5.** Specific information on protein annotation of four lysogenic phages.

**Additional file 2: Fig. S1.** Geographic information of contaminated sites and sampling locations. **Fig. S2.** Species richness of bacterial communities indicated by Chao1 index (A) and ACE index (B). **Fig. S3.** The phylogenetic tree of dominant bacterial species with relative abundance greater than 0.1%. **Fig. S4.** Profile of viral communities in Cr-contaminated soils. **Fig. S5.** This heat map indicates the distribution of top 20 viruses in different samples. **Fig. S6.** Redundant analysis (RDA) of virome. **Fig. S7.** The habitat composition of RefSeq viruses linked by viral contigs from Cr-contaminated soil. **Fig. S8.** Predicted virus-host linkages at the phylum level. **Fig. S9.** Predicted virus-host linkages at the genus level. **Fig. S10.** The non-metric multidimensional scaling (NMDS) of viral functional genes. **Fig. S11.** The relative abundance of virus-carried genes annotated by eggNOG database. **Fig. S12.** Epifluorescence-microscopy image of viruses.

## Data Availability

All raw sequence data generated in this research has been deposited in the Genome Sequence Archive (GSA) in BIG Data Center, Beijing Institute of Genomics, Chinese Academy of Sciences, under accession numbers CRA003088 and CRA003796, and are publicly accessible at https://bigd.big.ac.cn/gsa.

## Abbreviations

Cr: Chromium
AMG: Auxiliary metabolic gene
MRG: Heavy metal resistance gene
VC: Viral cluster
NCBI: National Center for Biotechnology Information
eggNOG: Evolutionary genealogy of genes: Non-supervised Orthologous Groups
KEGG: Kyoto Encyclopedia of Genes and Genomes
RDA: Redundant analysis
NMDS: Nonmetric multidimensional scaling
PCR: Polymerase chain reaction
WSOC: Water-soluble organic carbon

## References

[1] Martha RJC, Andrew DM, Andrey VL, Shaun H (2011). Phages in nature. Bacteriophage..

[2] Wang YL, Jiang XT, Liu L, Li B, Zhang T (2018). High-resolution temporal and spatial patterns of virome in wastewater treatment systems. Environ Sci Technol..

[3] Petrovich ML, Zilberman A, Kaplan A, Eliraz GR, Wang YB, Langenfeld K, Duhaime M, Wigginton K, Poretsky R, Avisar D, Wells GF (2020). Microbial and viral communities and their antibiotic resistance genes throughout a hospital wastewater treatment system. Front Microbiol..

[4] Cao B, Xu H, Mao CB (2014). Phage as a template to grow bone mineral nanocrystals. Methods Mol Biol..

[5] Suttle CA (2005). Viruses in the sea. Nature..

[6] Motlagh AM, Bhattacharjee AS, Coutinho FH, Dutilh BE, Casjens SR, Goel RK (2017). Insights of phage-host interaction in hypersaline ecosystem through metagenomics analyses. Front Microbiol..

[7] Manrique P, Dills M, Young MJ (2017). The human gut phage community and its implications for health and disease. Viruses-Basel..

[8] Pan D, Watson R, Wang D, Tan ZH, Snow DD, Weber K (2014). Correlation between viral production and carbon mineralization under nitrate-reducing conditions in aquifer sediment. Isme J..

[9] Nair RR, Vasse M, Wielgoss S, Sun L, Yu YTN, Velicer GJ (2019). Bacterial predator-prey coevolution accelerates genome evolution and selects on virulence-associated prey defences. Nat Commun..

[10] Kotay SM, Datta T, Choi JD, Goel R (2011). Biocontrol of biomass bulking caused by Haliscomenobacter hydrossis using a newly isolated lytic bacteriophage. Water Res..

[11] Calero-Caceres W, Ye M, Balcazar JL (2019). Bacteriophages as environmental reservoirs of antibiotic resistance. Trends Microbiol..

[12] Bhattacharjee AS, Choi J, Motlagh AM, Mukherji ST, Goel R (2015). Bacteriophage therapy for membrane biofouling in membrane bioreactors and antibiotic-resistant bacterial biofilms. Biotechnol Bioeng..

[13] Shkoporov AN, Hill C (2019). Bacteriophages of the human gut: the “known unknown” of the microbiome. Cell Host Microbe..

[14] Paez-Espino D, Eloe-Fadrosh EA, Pavlopoulos GA, Thomas AD, Huntemann M, Mikhailova N, Rubin E, Ivanova NN, Kyrpides NC (2016). Uncovering Earth’s virome. Nature..

[15] Rodriguez-Brito B, Li LL, Wegley L, Furlan M, Angly F, Breitbart M, Buchanan J, Desnues C, Dinsdale E, Edwards R, Felts B, Haynes M, Liu H, Lipson D, Mahaffy J, Martin-Cuadrado AB, Mira A, Nulton J, Pasic L, Rayhawk S, Rodriguez-Mueller J, Rodriguez-Valera F, Salamon P, Srinagesh S, Thingstad TF, Tran T, Thurber RV, Willner D, Youle M, Rohwer F (2010). Viral and microbial community dynamics in four aquatic environments. Isme J..

[16] Warwick-Dugdale J, Buchholz HH, Allen MJ, Temperton B (2019). Host-hijacking and planktonic piracy: how phages command the microbial high seas. Virol J..

[17] Scanlan PD, Hall AR, Blackshields G, Friman VP, Davis MR, Goldberg J, Joanna B, Buckling A (2015). Coevolution with bacteriophages drives genome-wide host evolution and constrains the acquisition of abiotic-beneficial mutations. Mol Biol Evol..

[18] Roossinck MJ (2011). The good viruses: viral mutualistic symbioses. Nat Rev Microbiol..

[19] Emerson JB, Roux S, Brum JR, Bolduc B, Woodcroft BJ, Jang HB, Singleton CM, Soden LM, Naas AE, Boyd JA, Hodgkins SB, Wilson RM, Trubl G, Li CS, Frokings S, Pope PB, Wrighton KC, Crill PM, Chanton JP, Saleska SR, Tyson GW, Rich VI, Sullivan MB (2018). Host-linked soil viral ecology along a permafrost thaw gradient. Nat Microbiol..

[20] Howard-Varona C, Hargreaves KR, Abedon ST, Sullivan MB (2017). Lysogeny in nature: mechanisms impact and ecology of temperate phages. Isme J..

[21] Zhang JY, Gao Q, Zhang QT, Wang TX, Yue HW, Wu LW, Shi J, Qin ZY, Zhou JZ, Zuo JE, Yang YF (2017). Bacteriophage-prokaryote dynamics and interaction within anaerobic digestion processes across time and space. Microbiome..

[22] Walker CB, Stolyar S, Chivian D, Pinel N, Gabster JA, Dehal PS, He ZL, Yang ZK, Yen HCB, Zhou JZ, Wall JD, Hazen TC, Arkin AP, Stahl DA (2009). Contribution of mobile genetic elements to Desulfovibrio vulgaris genome plasticity. Environ Microbiol..

[23] Maslov S, Sneppen K (2015). Well-temperate phage: optimal bet-hedging against local environmental collapses. Sci Rep-UK..

[24] Xu JW, Liu C, Hsu PC, Zhao J, Wu T, Tang J, Liu K, Cui Y (2019). Remediation of heavy hetal contaminated soil by asymmetrical alternating current electrochemistry. Nat Commun.

[25] Mielke HW, Gonzales CR, Powell ET, Laidlaw MAS, Berry KJ, Laidlaw MAS, Berry KJ, Mielke PW, Egendorf SP (2019). The concurrent decline of soil lead and children's blood lead in New Orleans. Proc Natl Acad Sci USA..

[26] Wu G, Kang HB, Zhang XY, Shao HB, Chu LY, Ruan CJ (2010). A critical review on the bio-removal of hazardous heavy metals from contaminated soils: issues progress eco-environmental concerns and opportunities. J Hazard Mater..

[27] Liu XY, Bai ZK, Shi HD, Zhou W, Liu XC (2019). Heavy metal pollution of soils from coal mines in China. Nat Hazards..

[28] Zou YD, Wang XX, Khan A, Wang PY, Liu YH, Alsaedi A, Hayat T, Wang XK (2016). Environmental remediation and application of nanoscale zero-valent iron and its composites for the removal of heavy metal ions: a review. Environ Sci Technol..

[29] Ma Y, Zhong H, He ZG (2019). Cr (VI) reductase activity locates in the cytoplasm of Aeribacillus pallidus BK1 a novel Cr(VI)-reducing thermophile isolated from Tengchong geothermal region China. Chem Eng J..

[30] Gao SM, Schippers A, Chen N, Yuan Y, Zhang MM, Li Q, Liao B, Shu WS, Huang LN (2020). Depth-related variability in viral communities in highly stratified sulfidic mine tailings. Microbiome..

[31] Trubl G, Jang HB, Roux S, Emerson JB, Solonenko N, Vik DR, Solden L, Ellenbogen J, Runyon AT, Bolduc B, Woodcroft BJ, Saleska SR, Tyson GW, Wrighton KC, Sullivan MB, Rich VI (2018). Soil viruses are underexplored players in ecosystem carbon processing. Msystems..

[32] Yu PF, Mathieu J, Yang Y, Alvarez PJJ (2017). Suppression of enteric bacteria by bacteriophages: importance of phage polyvalence in the presence of soil bacteria. Environ Sci Technol..

[33] Yu PF, Mathieu J, Lu GW, Gabiatti N, Alvarez PJ (2017). Control of antibiotic-resistant bacteria in activated sludge using polyvalent phages in conjunction with a production host. Environ Sci Tech Let..

[34] Yang JX, Wei W, Pi SS, Ma F, Li A, Wu D, Xing J (2015). Competitive adsorption of heavy metals by extracellular polymeric substances extracted from *Klebsiella sp J1*. Bioresource Technol..

[35] Dogan NM, Kantar C, Gulcan S, Dodge CJ, Yimaz BC, Mazmanci MA (2011). Chromium (VI) bioremoval by Pseudomonas bacteria: role of microbial exudates for natural attenuation and biotreatment of Cr (VI) contamination. Environ Sci Technol..

[36] Ng TW, Cai QH, Wong CK, Chow AT, Wong PK (2010). Simultaneous chromate reduction and azo dye decolourization by *Brevibacterium casei*: azo dye as electron donor for chromate reduction. J Hazard Mater..

[37] Anderson C, Jakobsson AM, Pedersen K (2007). Influence of in situ biofilm coverage on the radionuclide adsorption capacity of subsurface granite. Environ Sci Technol..

[38] Kumar R, Singh R, Kumar N, Bishnoi K, Bishnoi NR (2009). Response surface methodology approach for optimization of biosorption process for removal of Cr (VI), Ni (II) and Zn (II) ions by immobilized bacterial biomass *sp Bacillus brevis*. Chem Eng J..

[39] Lamont I, Richardson H, Carter DR, Egan JB (1993). Genes for the establishment and maintenance of lysogeny by the temperate coliphage 186. J Bacteriol..

[40] Villafane R, Black J (1994). Identification of 4 genes involved in the lysogenic pathway of the *Salmonella newington* bacterial-virus epsilon (34). Arch Virol..

[41] Yin H, He BY, Peng H, Ye JH, Yang F, Zhang N (2008). Removal of Cr (VI) and Ni (II) from aqueous solution by fused yeast: study of cations release and biosorption mechanism. J Hazard Mater..

[42] Nies DH (2003). Efflux-mediated heavy metal resistance in prokaryotes. Fems Microbiol Rev..

[43] Miao Y, Liao RH, Zhang XX, Wang Y, Wang Z, Shi P, Liu B, Li AM (2015). Metagenomic insights into Cr (VI) effect on microbial communities and functional genes of an expanded granular sludge bed reactor treating high-nitrate wastewater. Water Res..

[44] Malaviya P, Singh A. Metagenomic insights into Cr (VI) effect on microbial communities and functional genes of an expanded granular sludge bed reactor treating high-nitrate wastewater. Water Res. 2015;76:43-52. 10.1016/j.watres.2015.02.042 25792433

[45] Martinez-Garcia M, Santos F, Moreno-Paz M, Parro V, Anton JL (2014). Unveiling viral-host interactions within the ‘microbial dark matter’. Nat Commun..

[46] Forterre P (2013). The virocell concept and environmental microbiology. Isme J..

[47] Breitbart M, Bonnain C, Malki K, Sawaya NA (2018). Phage puppet masters of the marine microbial realm. Nat Microbiol..

[48] Dhal B, Thatoi HN, Das NN, Pandey BD (2013). Chemical and microbial remediation of hexavalent chromium from contaminated soil and mining/metallurgical solid waste: A review. J Hazard Mater..

[49] Liu YR, Delgado-Baquerizo M, Bi L, Zhu J, He JZ (2018). Consistent responses of soil microbial taxonomic and functional attributes to mercury pollution across China. Microbiome..

[50] Shapiro OH, Kushmaro A, Brenner A (2010). Bacteriophage predation regulates microbial abundance and diversity in a full-scale bioreactor treating industrial wastewater. Isme J..

[51] Liang XL, Zhang YY, Wommack KE, Wilhelm SW, DeBruyn JM, Sherfy AC, Zhuang J, Radosevich M (2020). Lysogenic reproductive strategies of viral communities vary with soil depth and are correlated with bacterial diversity. Soil Biol Biochem..

[52] Touchon M, de Sousa JAM, Rocha EPC (2017). Rocha Embracing the enemy: the diversification of microbial gene repertoires by phage-mediated horizontal gene transfer. Curr Opin Microbiol..

[53] Laslett D, Canback B (2004). ARAGORN, a program to detect tRNA genes and tmRNA genes in nucleotide sequences. Nucleic Acids Res..

[54] Yu PF, Mathieu J, Li MY, Dai ZY, Alvarez PJJ (2016). Isolation of polyvalent bacteriophages by sequential multiple-host approaches. Appl Environ Microb..

[55] Feiner R, Argov T, Rabinovich L, Sigal N, Borovok I, Herskovits AA (2015). A new perspective on lysogeny: prophages as active regulatory switches of bacteria. Nat Rev Microbiol..

[56] Wang XX, Kim Y, Ma Q, Hong SH, Pokusaeva K, Sturino JM, Wood TK (2010). Cryptic prophages help bacteria cope with adverse environments. Nat Commun..

[57] Moon K, Jeon JH, Kang IN, Park KS, Lee KY, Cha CJ, Lee SH, Cho JC (2020). Freshwater viral metagenome reveals novel and functional phage-borne antibiotic resistance genes. Microbiome..

[58] Oliver KM, Degnan PH, Hunter MS, Moran NA (2009). Bacteriophages encode factors required for protection in a symbiotic mutualism. Science..

[59] Lucas PPB, Aymé S, Witold K, Marie-Christine B, Lars HH, João CS, Laurent P. Impact of phages on soil bacterial communities and nitrogen availability under different assembly scenarios. Microbiome. 2020;8(1):52. 10.1186/s40168-020-00822-z PMC7137350 32252805

[60] Rowell DL (1994). Soil science: methods & applications.

[61] EPA US (1996). Alkaline digestion for hexavalent chromium (EPA Method 3060a).

[62] Patel A, Noble RT, Steele JA, Schwalbach MS, Hewson I, Fuhrman JA (2007). Virus and prokaryote enumeration from planktonic aquatic environments by epifluorescence microscopy with SYBR Green I. Nat Protoc..

[63] Chen SF, Zhou YQ, Chen YR, Gu J (2018). fastp: an ultra-fast all-in-one FASTQ preprocessor. Bioinformatics..

[64] Li DH, Luo RB, Liu CM, Leung CM, Ting HF, Sadakane K, Yamashita H, Lam TW (2016). MEGAHIT v1, 0: a fast and scalable metagenome assembler driven by advanced methodologies and community practices. Methods..

[65] Li H. Minimap2: pairwise alignment for nucleotide sequences. Bioinformatics. 2018;34(18):3094–3100. 10.1093/bioinformatics/bty191 PMC6137996 29750242

[66] Li H, Handsaker B, Wysoker A, Fennell T, Ruan J, Homer N, Marth G, Abecasis G, Durbin R. The Sequence Alignment/Map format and SAMtools, Bioinformatics. 2009;25(16):2078–9. 10.1093/bioinformatics/btp352 PMC2723002 19505943

[67] Huson DH, Auch AF, Qi J, Schuster SC. MEGAN analysis of metagenomic data. Genime Res.. 2007;17(3)377–86.. 10.1101/gr.5969107 PMC1800929 17255551

[68] Noguchi H, Taniguchi T, Itoh T (2008). Meta Gene Annotator: detecting species-specific patterns of ribosomal binding site for precise gene prediction in anonymous prokaryotic and phage genomes. DNA Res..

[69] Li WZ, Godzik A (2006). Cd-hit: a fast program for clustering and comparing large sets of protein or nucleotide sequences. Bioinformatics..

[70] Punta M, Coggill PC, Eberhardt RY, Mistry J, Tate J, Boursnell C, Pang N, Forslund K, Ceric G, Clements J, Heger A, Holm L, Sonnhammer ELL, Eddy SR, Bateman A, Finn RD (2012). The Pfam protein families database. Nucleic Acids Res..

[71] Kanehisa M, Sato Y, Morishima K (2016). BlastKOALA and GhostKOALA: KEGG tools for functional characterization of genome and metagenome sequences. J Mol Biol..

[72] Huerta-Cepas J, Szklarczyk D, Heller D, Hernandez-Plaza A, Forslund SK, Cook H, Mende DR, Letunic I, Rattei T, Jensen LJ, von Mering C, Bork P (2019). eggNOG 5, 0: a hierarchical functionally and phylogenetically annotated orthology resource based on 5090 organisms and 2502 viruses. Nucleic Acids Res..

[73] Menzel P, Ng KL, Krogh A (2016). Fast and sensitive taxonomic classification for metagenomics with Kaiju. Nat Commun..

[74] Adriaenssens EM, Kramer R, Van Goethem MW, Makhalanyane TP, Hogg I, Cowan DA (2017). Environmental drivers of viral community composition in Antarctic soils identified by viromics. Microbiome..

[75] Yu DT, He JZ, Zhang LM, Han LL (2019). Viral metagenomics analysis and eight novel viral genomes identified from the Dushanzi mud volcanic soil in Xinjiang China. J Soil Sediment..

[76] Fuhrman JA (1999). Marine viruses and their biogeochemical and ecological effects. Nature..

[77] Roux S, Hallam SJ, Woyke T, Sullivan MB (2015). Viral dark matter and virus-host interactions resolved from publicly available microbial genomes. Elife..

[78] Roux S, Enault F, Hurwitz BL, Sullivan MB (2015). VirSorter: mining viral signal from microbial genomic data. Peer J..

[79] De Sanctis M, Fanelli G, Gjeta E, Mullaj A, Attorre F (2018). The forest communities of Shebenik-Jabllanice National Park (Central Albania). Phytocoenologia..

