# Supplementary material for: Enhanced mutualistic symbiosis between soil phages and bacteria with elevated chromium-induced environmental stress
Source: Microbiome. 2021 Jun 28;9:150. doi: 10.1186/s40168-021-01074-1 (PMC8240259; doi:10.1186/s40168-021-01074-1)
Supplement: Supplementary file 3 — Additional file 2: Fig. S1. Geographic information of contaminated sites and sampling locations. Fig. S2. Species richness of bacterial communities indicated by Chao1 index (A) and ACE index (B). Fig. S3. The phylogenetic tree of dominant bacterial species with relative abundance greater than 0.1%. Fig. S4. Profile of viral communities in Cr-contaminated soils. Fig. S5. This heat map indicates the distribution of top 20 viruses in different samples. Fig. S6. Redundant analysis (RDA) of virome. Fig. S7. The habitat composition of RefSeq viruses linked by viral contigs from Cr-contaminated soil. Fig. S8. Predicted virus-host linkages at the phylum level. Fig. S9. Predicted virus-host linkages at the genus level. Fig. S10. The non-metric multidimensional scaling (NMDS) of viral functional genes. Fig. S11. The relative abundance of virus-carried genes annotated by eggNOG database. Fig. S12. Epifluorescence-microscopy image of viruses. [file 40168_2021_1074_MOESM3_ESM.docx]

**Supporting Information**

**Enhanced Mutualistic Symbiosis between Soil Phages and Bacteria with Elevated Chromium-Induced Environmental Stress**

Dan Huang^1, 2^, Pingfeng Yu^3, *^, Mao Ye^1, *^, Cory Schwarz^3^, Xin Jiang^1^, Pedro J.J. Alvarez^3^

^1^ Key Laboratory of Soil Environment and Pollution Remediation, Institute of Soil Science, Chinese Academy of Sciences, Nanjing 210008, PR China

^2^ University of Chinese Academy of Sciences, Beijing 100049, PR China

^3^ Department of Civil and Environmental Engineering, Rice University, Houston, 77005, United States

^*^ Corresponding Authors: [pingfeng.yu@rice.edu](mailto:pingfeng.yu@rice.edu) (P. Yu), yemao@issas.ac.cn (M. Ye)

**This** **supporting information includes 12 figures.**

Fig. S1. Geographic information of contaminated sites and sampling locations.

Fig. S2. Species richness of bacterial communities indicated by Chao1 index (A) and ACE index (B).

Fig. S3. Redundant analysis (RDA) of bacteria

Fig. S4. Profile of viral communities in Cr-contaminated soils.

Fig. S5. This heat map indicates the distribution of top 20 viruses in different samples.

Fig. S6. Redundant analysis (RDA) of virome.

Fig. S7. The habitat composition of RefSeq viruses linked by viral contigs from Cr-contaminated soil.

Fig. S8. Predicted virus-host linkages at the phylum level.

Fig. S9. Predicted virus-host linkages at the genus level.

Fig. S10. The non-metric multidimensional scaling (NMDS) of viral functional genes.

Fig. S11. The relative abundance of virus-carried genes annotated by eggNOG database.

Fig. S12. Epifluorescence-microscopy image of viruses.

Fig. S13. Transmission electron microscopic images of viral particles.

Fig. S14. Experimental procedure.


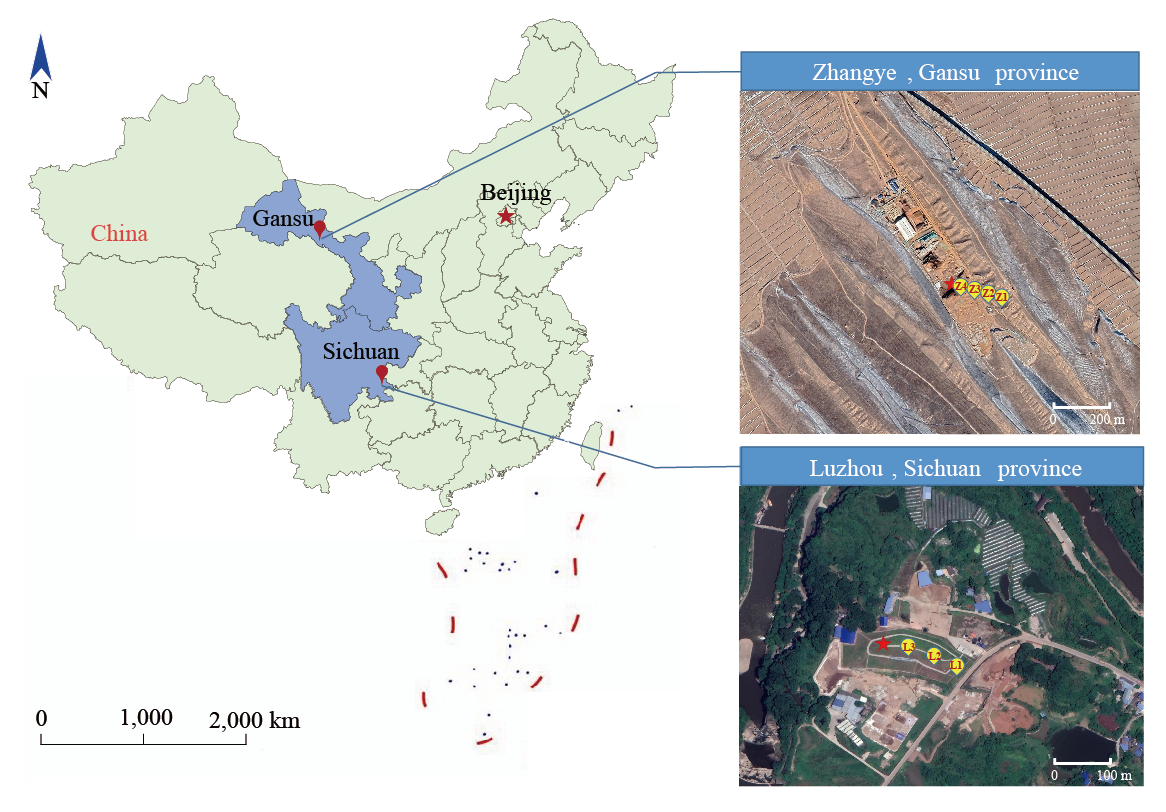


**Fig. S1.** Geographic information of contaminated sites and sampling locations. We investigated two chromium (Cr)-slag contaminated sites, located in Luzhou, Sichuan Province (LZ site, N 28° 54′ 24″, E 105° 31′ 26″) and Zhangye, Gansu Province (ZY site, N 38° 25′ 49″ E 100° 48′ 41″) in China. Soil samples were collected at intervals of 50 meters, from the Cr-slag dump center towards the edge of two sites. According to the site area, 3 soil samples (L1, L2, L3) were collected in LZ and 4 soil samples (Z1, Z2, Z3, Z4) were collected in ZY. Each soil sample was a manually homogenized composite of five subsamples collected at the surface layer (5-10 cm) and sieved through a size 20 mesh.


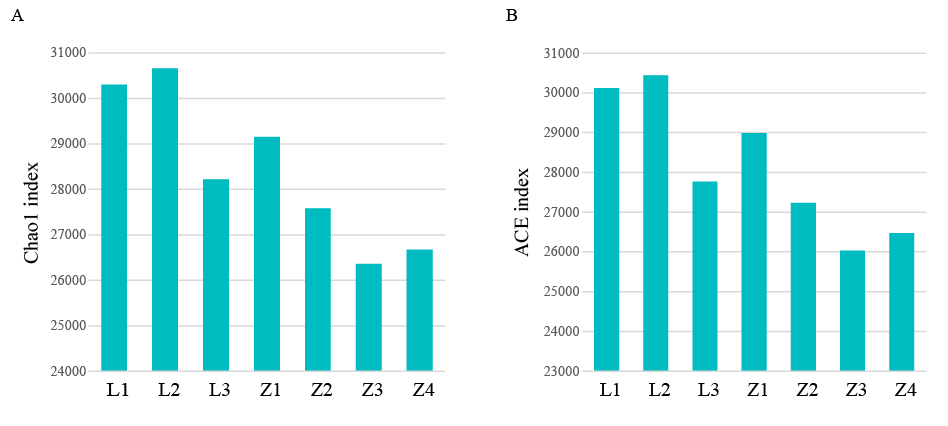


**Fig. S2.** Species richness of bacterial communities indicated by Chao1 index (A) and ACE index (B).


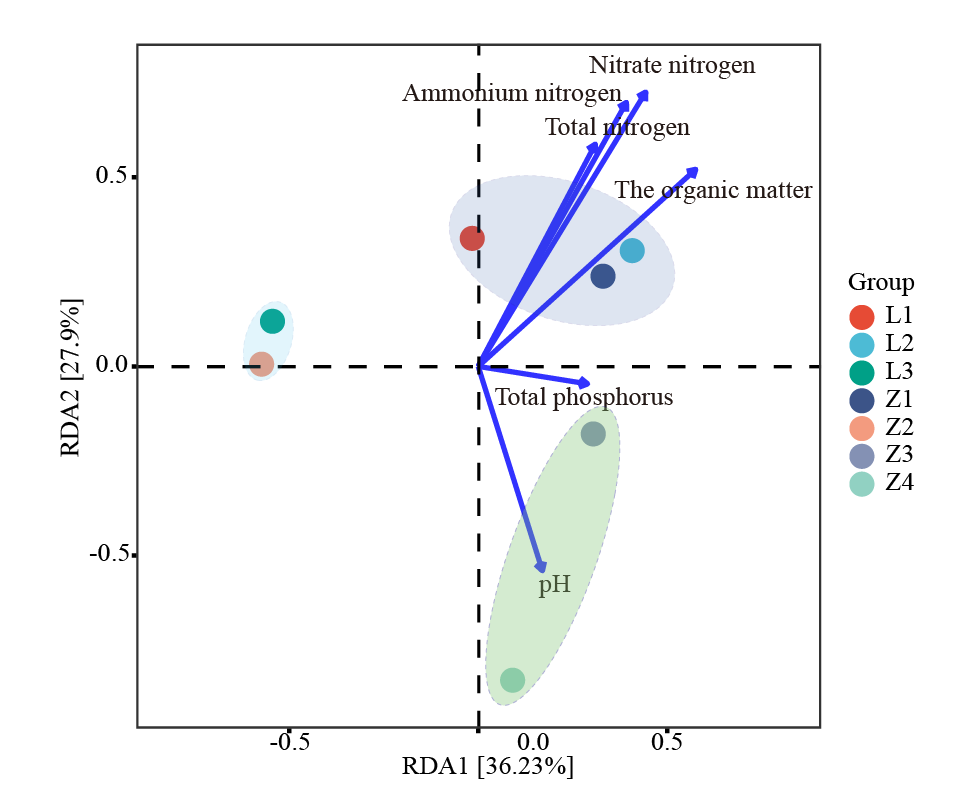


**Fig. S3.** Redundant analysis (RDA) of bacteria in different soil samples. The results showed nitrate, ammonium, total nitrogen, organic matter and total phosphorus had the closest correlation with the composition of bacteria. The bacterial communities of each sample clustered most closely with samples under similar geochemical conditions. . Specifically, L1, L2 and Z1 with low available Cr concentration were more similar, and L3 and Z2 with slightly higher pollution levels were clustered together. Z3 and Z4 were dramatically different from those in other sites.


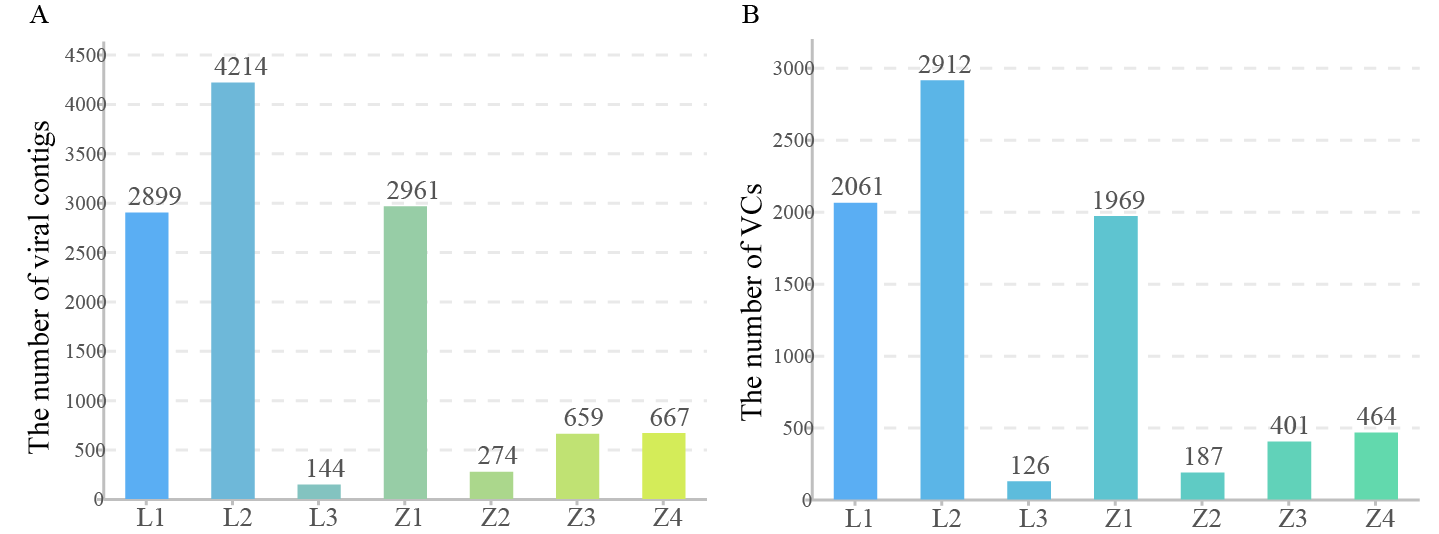


**Fig. S4.** Number of viral contigs (A) and clusters (B) of viral communities inCr-contaminated soils.


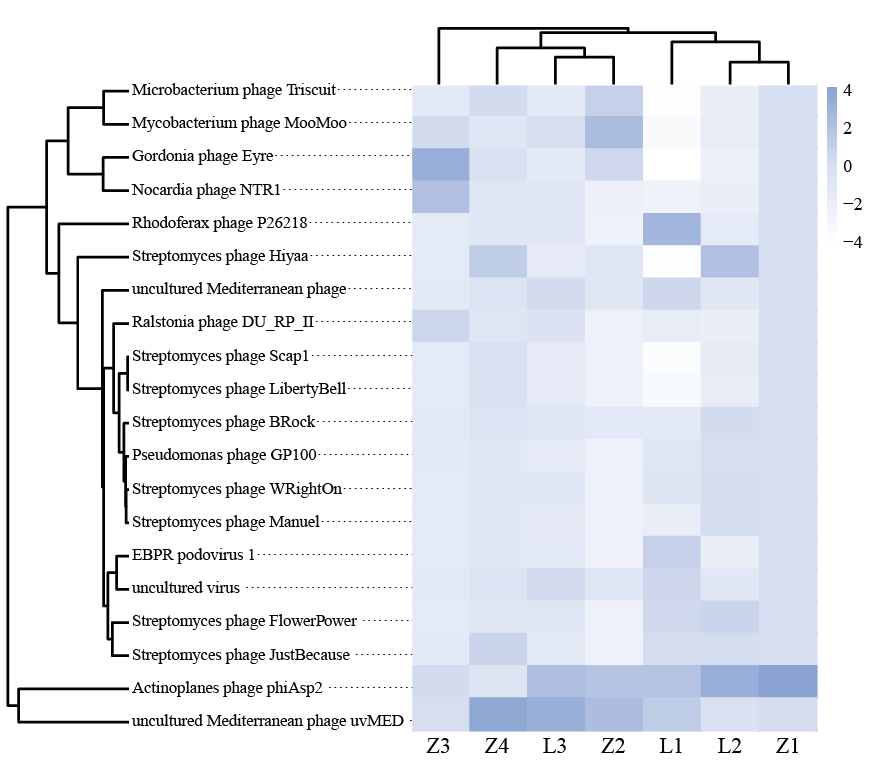


**Fig. S5.** This heat map indicates the distribution of top 20 viruses in different samples. Viruses with high relative abundance are indicated by purplish-blue colors, white represents lowest relative abundance levels. Viruses and soil samples were grouped by Ward. D method, showing viral populations in locations with similar pollution levels are more closely related.


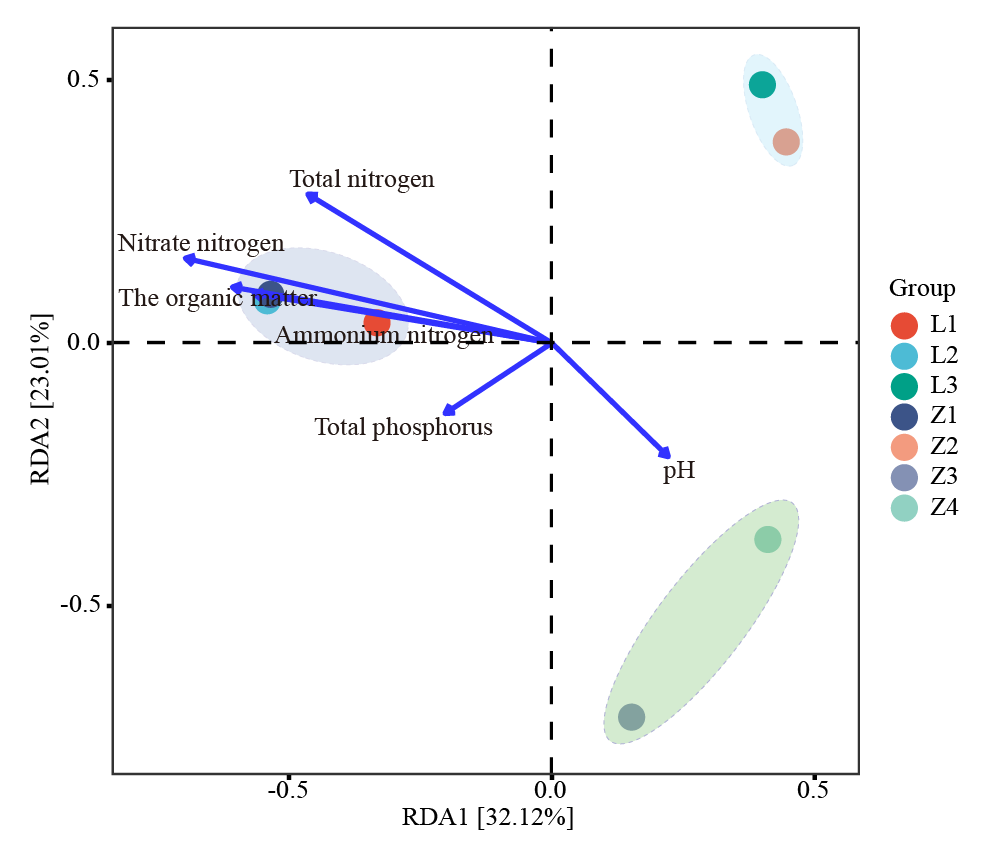


**Fig. S6.** Redundant analysis (RDA) of viromes from different soil samples. The results showed nitrate, ammonium, total nitrogen, organic matter and total phosphorus had the closest correlation with the composition of the viromes. The viromes of each sample clustered most closely with samples under similar geochemical conditions. Specifically, L1, L2 and Z1 with low available Cr concentration were more similar, and L3 and Z2 with slightly higher pollution levels were clustered together. Z3 and Z4 were dramatically different from those in other sites.


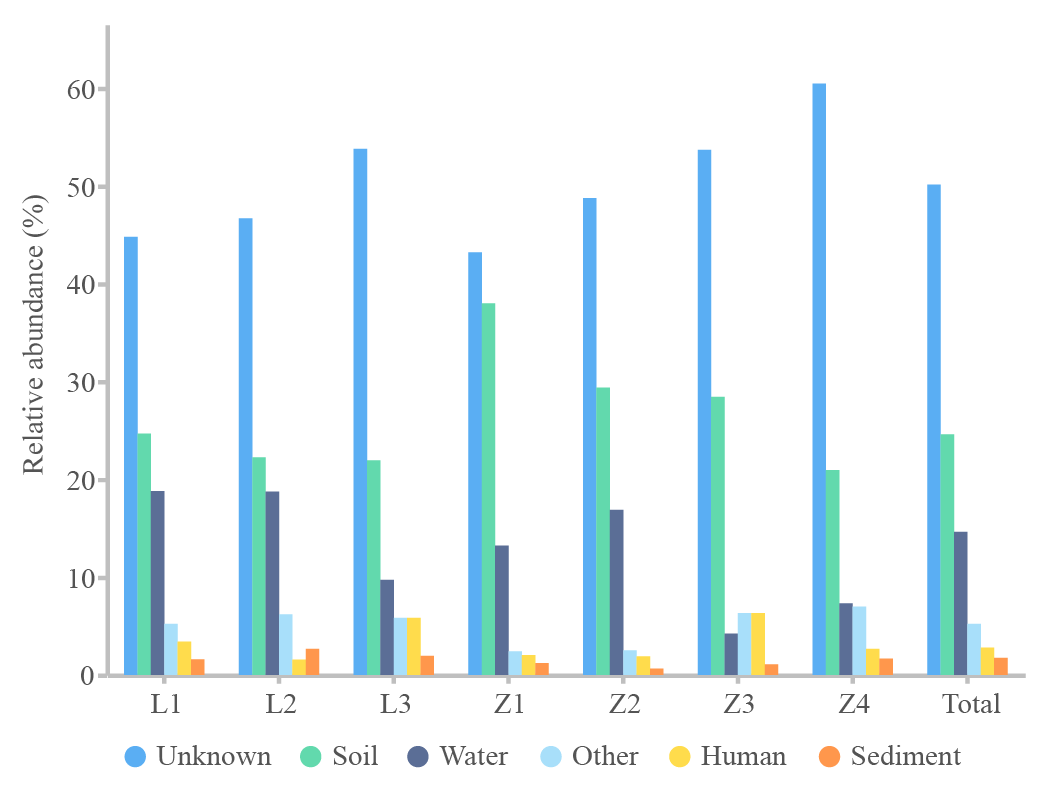


**Fig. S7.** The habitat composition of RefSeq viruses linked by viral contigs from Cr-contaminated soil.


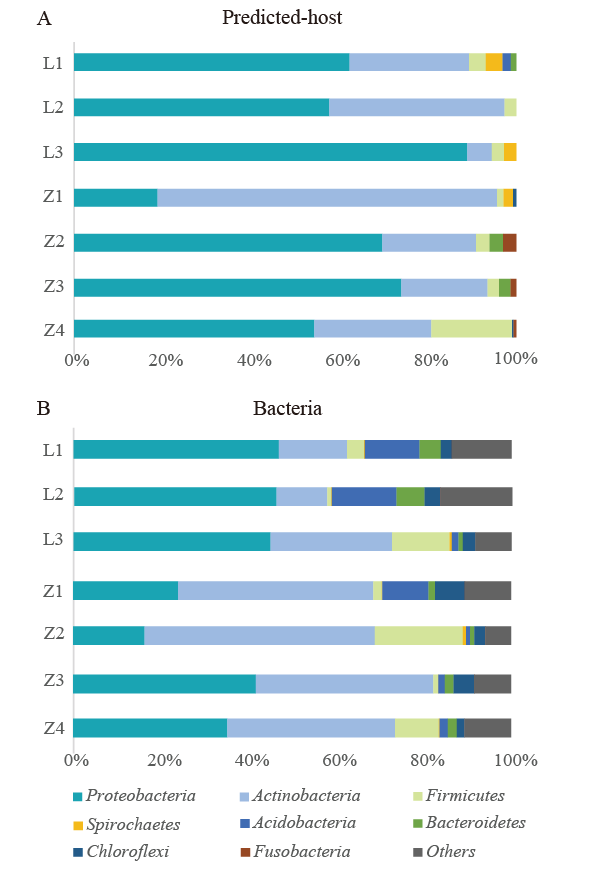


**Fig. S8**. Predicted virus-host linkages at the phylum level. The relative abundance distribution of predicted viral-host (A) and corresponding to the bacteria revealed by metagenomic analysis (B). The matched 279 host genera were mainly from eight bacterial phyla, most of which also accounted for the most abundant phyla in the bacterial communities.


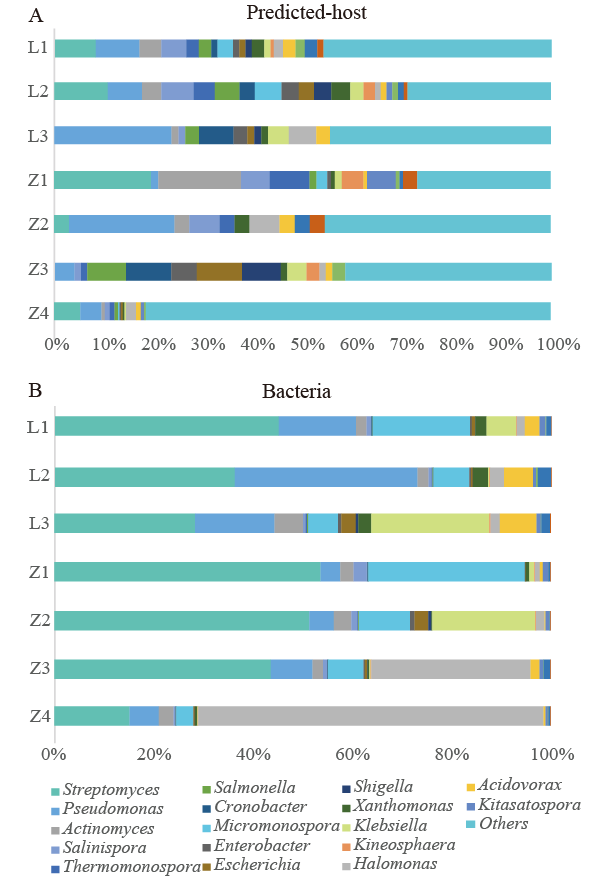


**Fig. S9**. Predicted virus-host linkages at the genus level. The relative abundance distribution of predicted viral-host (C) and the corresponding to top 20 bacterial genera in the metagenome (D). *Pseudomonas*, *Cronobacter*, *Klebsiella* and *Halomona* genera increased with available Cr concentration under moderate contamination conditions (up to 6.76 mg/kg). The increased four predicted viral host bacterial genera in Z3 were *Salmonella*, *Cronobacter, Enterobacter*, and in Z4 were *Bacillus*, *Stenotrophomonas*, *Comamonas* and *Lysinibacillus.*


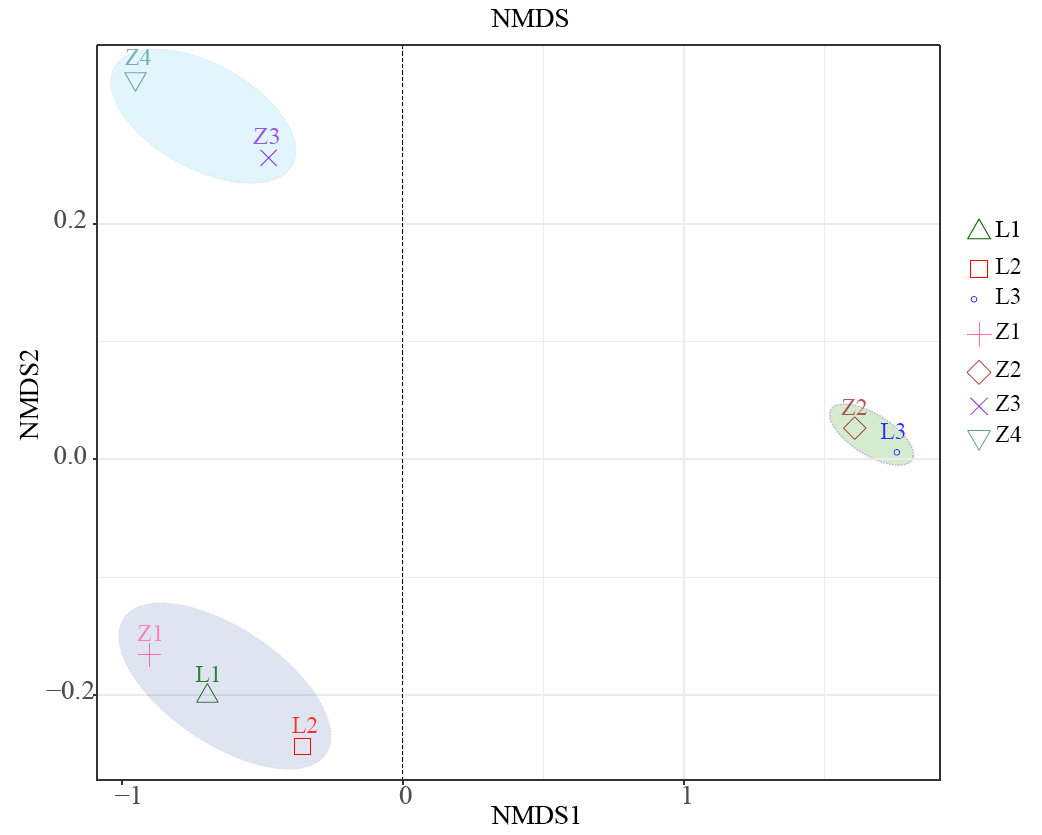


**Fig. S10**. The non-metric multidimensional scaling (NMDS) of functional genes was annotated by KEGG datasets based on Bray-Curtis distance. The viral contigs from samples with similar Cr concentrations were more similar in genetic compositions despite the spatial distance.


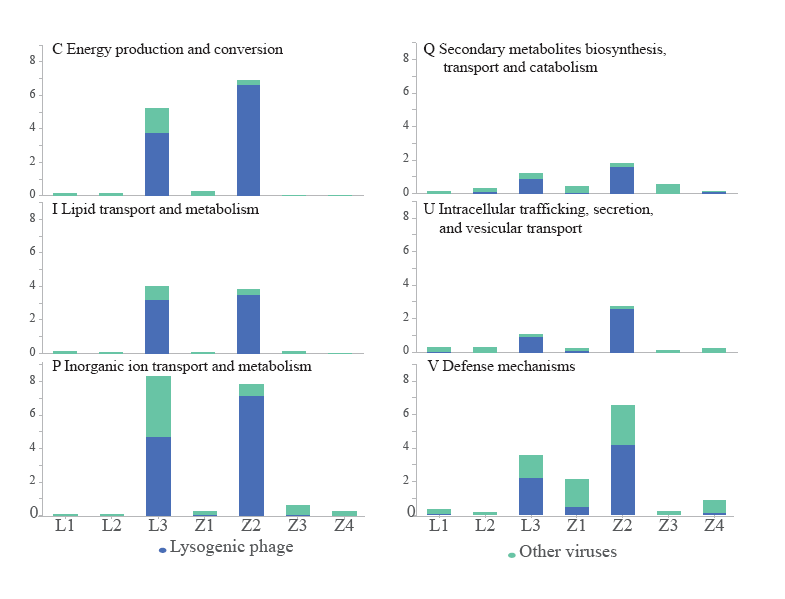


**Fig. S11**. The relative abundance of virus-carried genes annotated by eggNOG database, including C, I, P, Q, U and V6 involved in energy metabolism and environmental tolerance of microorganism. Blue indicates genes located in the genome of lysogenic phages, while green indicates genes located in that of other viruses. In L3 and Z2, the relative abundance of these genes was significantly increased, and a significant portion was observed to be carried by lysogenic phages.


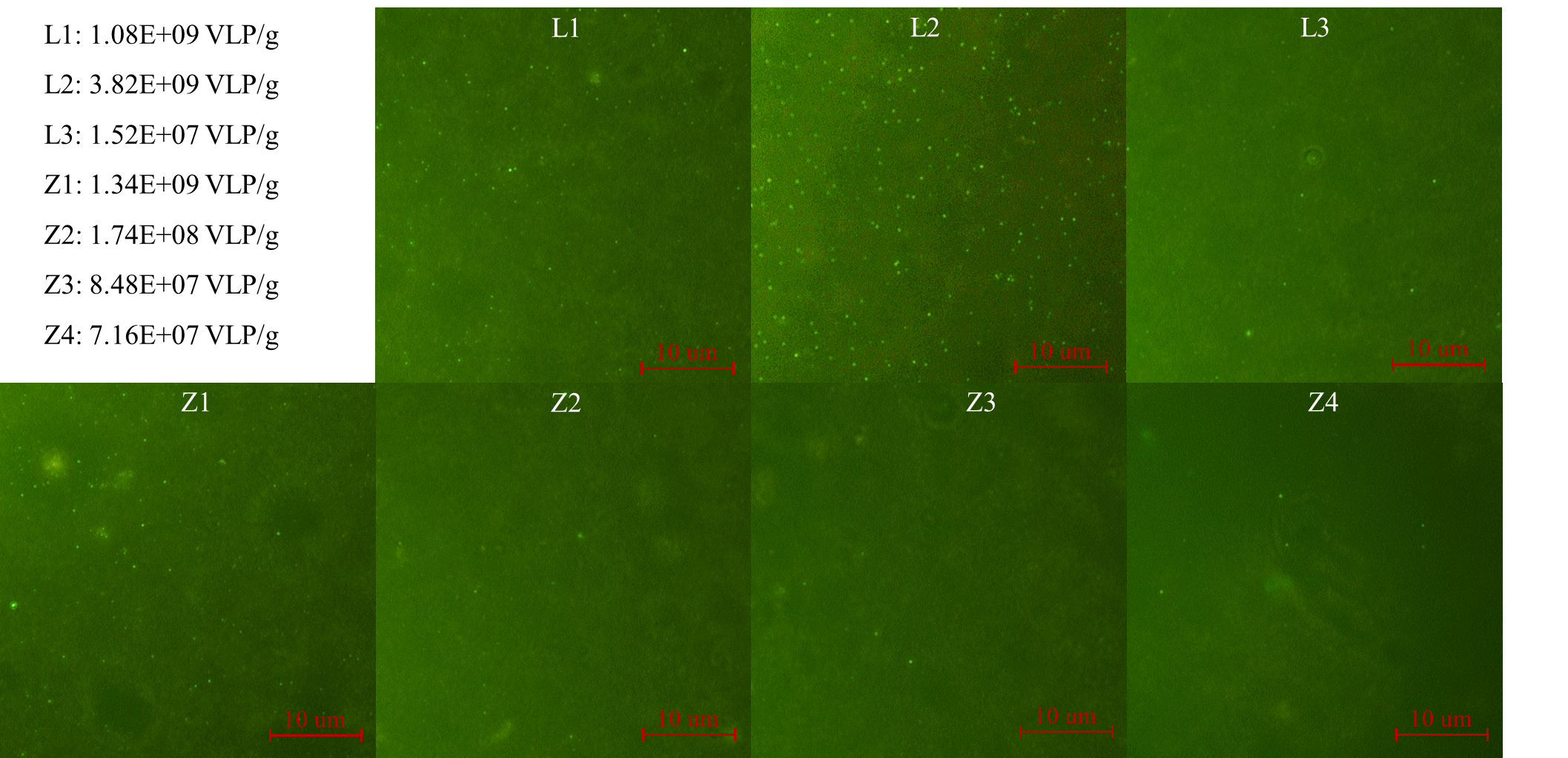
**Fig. S12.** Epifluorescence-microscopy image of viruses from a soil sample filtered onto a Whatman 0.02 mm Anodisc filter stained with SYBR Gold.


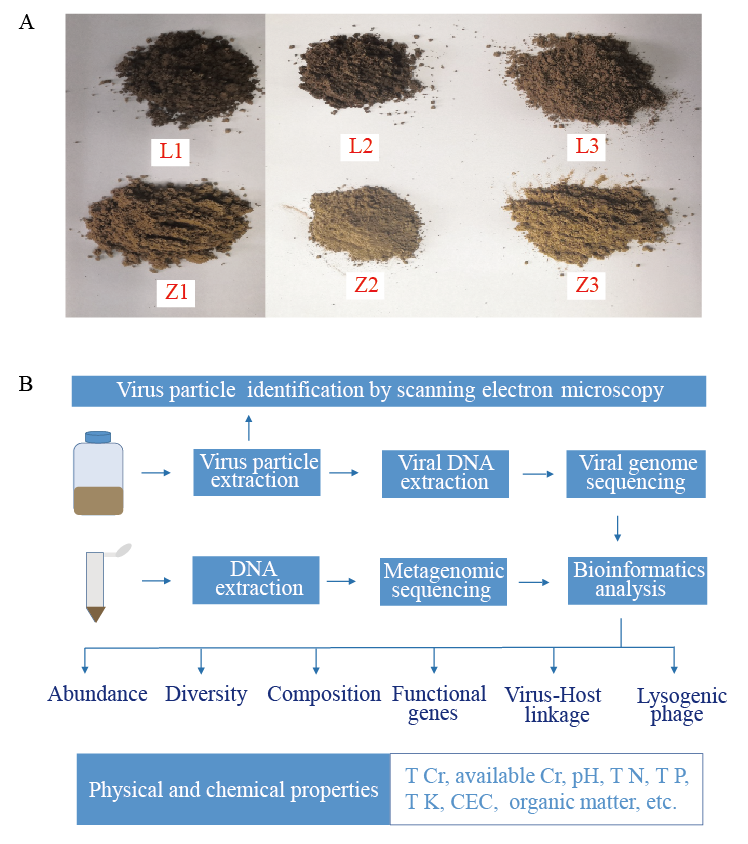


**Fig. S13.** Experimental procedure. The experimental procedure includes the recovery and morphological identification of virus particles, virome metagenomic and metagenomic sequencing and data analysis, and the analysis of physical and chemical properties of the soils.
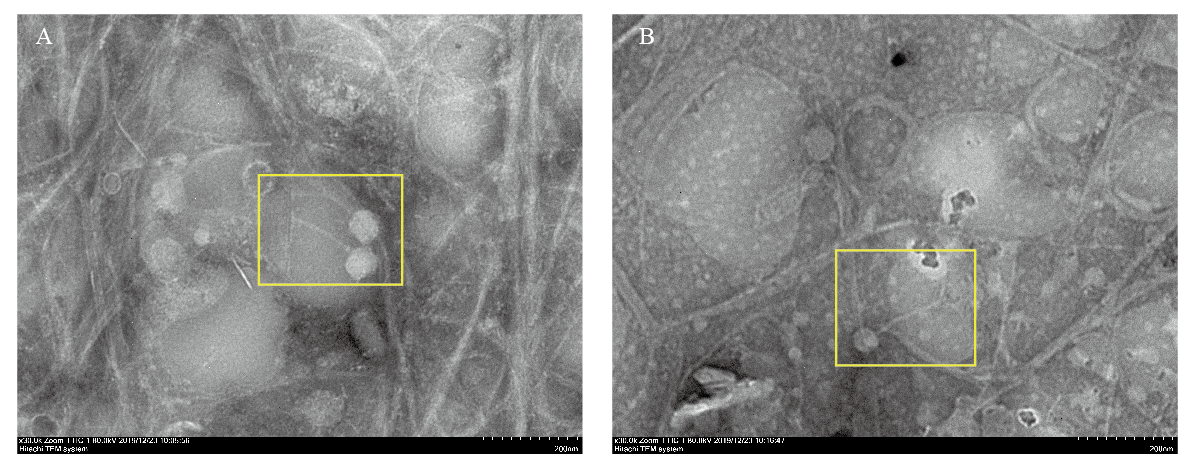


**Fig. S14.** Transmission electron microscopic images of viral particles extracted from Cr-contaminated soils. (A) is for LZ site located in Luzhou City, and (B) is for ZY site located in Zhangye City.
